# Scoping of Policy Impacts for Regulating E-cigarettes (SPIRE): findings from a data and decision analytic model mapping project

**DOI:** 10.3310/nihropenres.14038.1

**Published:** 2025-10-06

**Authors:** Hazel Squires, Duncan Gillespie, Loren Kock, Emma Hock, Rosemary Hiscock, Ilze Bogdanovica, Calum Lewis, Deborah Robson, Filippos Filippidis, Tessa Langley, Emily Pulsford, Mark Clowes, Sarah Jackson, Ann McNeill, John Mehegan, Anthony Laverty, Iona Fitzpatrick, Alan Brennan, Robin Purshouse, Jamie Brown, Lion Shahab

**Affiliations:** 1 The University of Sheffield, Sheffield, England, UK; 2 Behavioural Science and Health, University College London, London, England, UK; 3 University of Bath, Bath, England, UK; 4 University of Nottingham, Nottingham, England, UK; 5 King's College London School of Academic Psychiatry, London, England, UK; 6 Imperial College London School of Public Health, London, England, UK

**Keywords:** e-cigarette, nicotine, tobacco, model, data mapping, policy, UK, data synthesis

## Abstract

**Background:**

E-cigarettes, also known as vapes, are lower risk products compared to conventional cigarettes, that can aid smoking cessation. However, they have been developed to also appeal to people who do not smoke, and are not harm-free. The challenge is for vaping policy to support smokers to quit whilst also protecting non-smokers from starting. Simulation modelling can be used to synthesise existing evidence and make predictions about policy impacts. This research aims to identify (a) data sources that can inform modelling of vape policies in the United Kingdom (UK) and (b) gaps in data that are required to undertake appropriate modelling.

**Methods:**

We held stakeholder workshops with academic experts, policy makers and public members to understand the requirements of a simulation model of vaping policy and existing data. Based on the findings of the first workshop and a review of existing modelling studies, we undertook a set of targeted rapid reviews to augment key existing reviews. We also developed a dataset dictionary. From these, we developed key recommendations about data collection and modelling.

**Results:**

There is substantial UK evidence around many of the transitions between smoking and vaping behaviours, but these have not yet been estimated simultaneously. We also identified 25 UK studies assessing the socioeconomic, psychological and social network influences on vaping behaviours. However, there is limited evidence about the effectiveness of vaping policies in the UK, the impact of industry circumvention, the health harms of vaping for people who have never smoked, longer term evidence on the smoking harms of vaping and the use and impact of illegal vapes.

**Conclusions:**

Addressing the identified gaps in the evidence will require targeted new research. By fostering collaboration across disciplines and ensuring transparency and consistency in modelling, the UK can build a credible, evidence-based foundation for shaping effective vape regulation.

## Plain English Summary

E-cigarettes, or vapes, are less harmful than traditional cigarettes and can help people quit smoking. However, they are not risk-free and may attract people who have never smoked. This presents a challenge: how can vaping policies both support smokers to quit while also protecting non-smokers from starting?

One way to explore this is through simulation modelling—a computer-based method that combines existing evidence to test “what if” scenarios. For example, what might happen to smoking and vaping rates if new restrictions on vape flavours were introduced? These models can help predict outcomes before policies are implemented in real life. But to make them accurate, we need good data.

This project aimed to identify 1) What UK data already exist to inform vape policy modelling; 2) Where the gaps are and what new data are needed.

We held workshops with researchers, policy makers, and the public to agree on what a useful model should include. We reviewed existing modelling studies and datasets and filled in evidence gaps with focused literature reviews. From this, we developed a “data dictionary”—a guide to the most useful UK data—and made recommendations to improve data collection and modelling.

We found lots of UK data on how people move between smoking and vaping (e.g., quitting smoking by vaping or starting to smoke after vaping), but these behaviours have not yet been modelled together. We also found 25 UK studies on how social, economic, and psychological factors affect vaping. However, key gaps remain: there is limited UK data on how specific policies work, on illegal vape use and its impact, how the industry responds to rules and what the long-term health risks are (especially for never-smokers).

We recommend targeted studies and better coordination across research and policy to build stronger, more useful evidence for future regulations.

## 1. Introduction

E-cigarettes, also known - and referred to in this article - as vapes, are considered lower risk products compared to conventional cigarettes ^1^ that can aid smoking cessation ^2^. Over the last decade vapes have been a popular and effective stop smoking aid in the United Kingdom (UK) ^3^. However, vapes have also been developed to appeal to people who do not smoke, particularly to young people, for example via flavours, packaging, low prices and the availability of single use (disposable) products ^4^. Other modifications in vape products, such as the increasing use of nicotine salts, likely increase their addiction potential via more effective nicotine delivery ^5, 6^. The potential impacts of vaping on health (including long-term physical harms and consequences of addiction, especially among people who have never smoked) and the impact of changing policies to regulate these products on the use of both vapes and tobacco are currently uncertain. Appraisal of the different policy options would benefit from computer modelling that integrates and analyses data from multiple sources to understand how various policy options related to vapes, for example the ban on disposable vapes, are likely to affect use of vapes and tobacco products overall and in different groups, for example young people. These models can also test the impact of different assumptions and data points where the evidence is uncertain. However, we do not currently have a good overview of what data are available to model the impact of vaping policies in the UK or know what further data collection should be prioritised to improve such modelling.

Previous work has been undertaken to identify UK research priorities for vapes ^7, 8^; however, this has not had a focus on data availability and how to model possible policy impacts.

### 1.1 Aim of the research

This research aims to identify (a) data sources that can inform modelling of priority vape policies and (b) gaps in data that are required to undertake appropriate modelling by:

1. Engaging with stakeholders to identify priority vape policy options, mechanisms of action, unintended consequences, key subgroups and outcomes of interest;
2. Providing recommendations for the types of modelling that could be constructed to assess the impact of key vape policies;
3. Establishing which categories of data would be most valuable and at what level of detail;
4. Identifying data currently available and describing any potential issues including data accessibility and access costs;
5. Suggesting new types of data that would need to be collected to allow accurate modelling of vape policies and how future research efforts could be coordinated.

## 2. Methods

**Figure 1.**
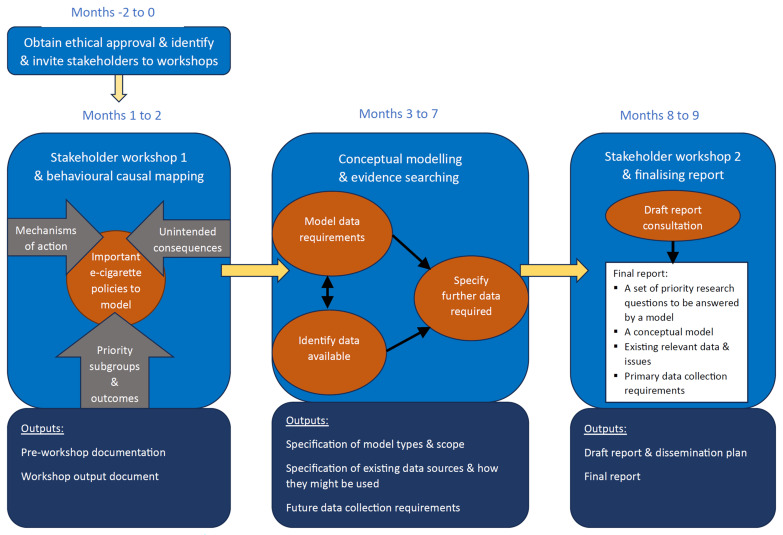
Project overview.

We followed a framework by Squires *et al.* ^9^ for developing the structure of public health models. This sets out four key principles of good practice in developing valid, credible and feasible models of public health interventions. These are: (1) a systems approach - a holistic way of thinking about the interactions between parts in a system and its environment - to public health modelling; (2) a documented understanding of the problem before and alongside developing and justifying the model structure; (3) strong communication with stakeholders throughout model development; and (4) a systematic consideration of the determinants of health to identifying key impacts of the interventions ^9^. Figure 1 provides an overview of the steps taken to produce this article, described in more detail below. Ethical approval for this project was provided by the University of Sheffield (reference number 064374). All participants in the workshops provided written informed consent to participate.

### 2.1 Stakeholder input

We identified a broad range of UK based experts who could provide advice about different aspects of the vaping policy system. These included key policy makers/ policy enforcers (e.g., Department of Health and Social Care, Devolved Governments, Office for Health Improvement and Disparities, HMRC, Trading Standards), non-governmental policy experts (e.g., Action on Smoking and Health England), public members, and experts in public health, behavioural science, data collection/analysis and modelling methods. Note that there were no stakeholders with experience in waste management. A list of all stakeholders involved in the workshops is available at https://osf.io/8zaxc/.

We held an in-person stakeholder workshop on 7th November 2024 which aimed to understand vaping policy options that could be effectively evaluated through modelling in the UK context, explore how these policies might influence behaviour, unintended effects of the policies and which groups of the population might be most at risk of harm or benefit from these policies. There were 46 attendees at the workshop, from which we produced a workshop outcomes document which was sent to all attendees for review before being finalised.

Through conceptual modelling (Sections 3 – Sections 6) and evidence identification (Section 7), we developed a draft of this report, which was sent to all stakeholders for review, as well as ten newly invited international academic stakeholders (from Australia, Canada, Italy, Netherlands, Switzerland and USA). We then held a second workshop on 3rd June 2025 which aimed to discuss and resolve any key issues arising from the review of the draft report, to evaluate whether the proposed approach and data collection would align with stakeholders’ current or anticipated information needs, to assess utility and generalisability to non-UK contexts and to plan dissemination and next steps. We held a third online workshop with international stakeholders who could not attend the second workshop due to large time difference (American and Australian colleagues) on 10th June 2025 (GMT).

### 2.2 Patient and Public Involvement

Involvement of lay people (Patient and Public Involvement and Engagement (PPIE)) was a core component of the SPIRE project, ensuring that the research was relevant and responsive to the needs and perspectives of patients and the public. The primary aim was to integrate lived experience into all stages of the study, from design to dissemination.

We adopted a co-creation approach, actively involving six PPIE partners: four recruited from existing PPIE panels at the Universities of Nottingham and Stirling, and two UCL university students included to ensure age diversity. PPIE partners participated across all phases of the project, including study design, policy option development, interpretation of findings, and dissemination planning.

At the outset, an online training session introduced PPIE partners to the project’s aims, methods (including systems mapping and policy analysis), and policy context. Their feedback shaped the initial development of policy options that were discussed at the first project workshop. PPIE partners attended the first in-person workshop, contributing to the refinement of behavioural systems maps and identification of priority areas alongside key policy and other stakeholders. They also participated in the second workshop to interpret findings and co-develop dissemination strategies, and contributed to developing the plain English summary. The group facilitators ensured that everyone was able to contribute to each session, including the PPIE.

The PPIE partners were an integral part of the workshop groups. PPIE involvement enhanced the study’s relevance, ethical robustness, and potential policy impact. The consistent engagement of a small, diverse group enabled deeper contextual insight and improved the accessibility and credibility of outputs. While socio-economic diversity could not be formally assessed due to the sensitive nature of the topic, the approach fostered trust, continuity, and meaningful integration of public perspectives into the research process.

### 2.3 Evidence identification

***2.3.1 Dataset dictionary***

 Before, during and after the first workshop we asked stakeholders to identify UK datasets that may be helpful for modelling vape policies. Following the workshop, we developed a spreadsheet to capture the characteristics (including design and accessibility) and information available within each of these datasets relevant to vape policy modelling.

***2.3.2 Literature reviews***

To assess the suitability of existing models of vaping policies, their strengths and weaknesses, the types of evidence used and key drivers of model results, we reviewed existing simulation modelling studies of vaping policies developed worldwide. We utilised two existing systematic reviews of models of vaping policies ^10, 11^ and also identified two models specific to the UK ^12, 13^.

As part of the first stakeholder workshop process, we identified some key vape evidence reviews, and we identified important evidence for modelling for which the evidence base was unknown or uncertain. Based on the outcomes of the first workshop and the existing modelling studies, we agreed upon three key review questions that would be important to feed into any modelling work, but for which there were not already key evidence reviews published. These questions were:

**Review question 1:** What are the health and wellbeing impacts of vaping on people who have never smoked in the UK?

**Review question 2:** Three questions on transitions between behaviours in the UK context:

a) What are the transition probabilities between smoking and vaping behaviours? (With a particular focus on whether this differs for (i) young people, (ii) people from different socioeconomic status (SES) groups or (iii) people with mental health issues)
b) What are the relative impacts of priority policies on these transitions? (With a particular focus on the variation in the impact of policy between: (i) young people, (ii) people from different SES groups, (iii) people with mental health issues, (iv) people who smoke and (v) people who have quit smoking)
c) What is the evidence for social or individual behavioural influences on the transitions between vaping behaviours?

**Review question 3:** Two questions on industry responses to priority government policies (to assess which of these reactions are currently or potentially modellable and, if so, how these can be modelled):

a) How has vaping industry reacted to government policies?
b) What impact has this had on the effectiveness of these government policies?

Review questions 1 and 2 were limited to the UK because health and transition implications may be reliant on the UK’s particular population profile, social norms about smoking and vaping, and the legal framework and the characteristics (e.g., ingredients) of the products available on the UK market (e.g., implementation of the revised Tobacco Products Directive into law in 2016 placed restrictions on some products).

Comprehensive searches were undertaken for published literature using MEDLINE, Embase, PsycINFO, CINAHL, The Cochrane Library and Web of Science. Due to the expected overlap between review questions, a single search strategy was designed to cover all review questions. The strategy was developed for MEDLINE (accessed via Ovid), and then adapted as appropriate for the other databases. The searches were performed simultaneously on the databases between 10th and 14th February 2025. Results were deduplicated in EndNote and then exported into Rayyan for screening for eligibility for any of the review questions. Results were limited to articles in the English language and published since January 2016 (to coincide with when UK legislation on vapes first came into force ^14^).

The search strategy was developed to include free-text search terms, and database-specific subject headings (e.g., MeSH, Emtree) where applicable, based around the following concepts: ‘vaping’, ‘vapes’; ‘harms’; ‘non-smokers’, ‘never smokers’; words to reflect potential transitions between smoking statuses, e.g., ‘initiation’, ‘dual use’, ‘cessation’; words to capture vaping-related policies (e.g., ‘flavour bans’, ‘health warnings’, ‘taxes’) and industry responses to such policies. Terms to limit to the UK were applied using the published and validated NICE filter in full where possible (in the case of MEDLINE ^15^ and Embase ^16^) or in adapted form in the case of the other databases. These were then combined with other search concepts using Boolean operators where needed (to cover review questions 1 and 2). The search strategy is available at https://osf.io/8zaxc/.

For all review questions we excluded studies affiliated with the tobacco or vaping industry (including study sponsors, any funding, author affiliations or conflicts of interest) to avoid bias. A table of all excluded studies is available at https://osf.io/8zaxc/.

For Review question 1, we included studies examining the impact of medium or long-term exposure (i.e., more than experimental use) to vapes (any type of device, excluding heated tobacco products) on health impacts (measured quantitatively) among people who have never smoked (a pragmatic definition, allowing for those who may have experimented with one or two cigarettes in the past) in the UK. We excluded studies that did not specify the country or smoking status.

For Review question 2a, we included studies that reported on the probability of transitioning between paired smoking and vaping states among the general population in the UK (with a focus on the following populations where the data exist: (i) young people (aged ≤25 years), (ii) people from different SES groups or (iii) people with mental health issues) in the UK. For review sub-question 2(b), we included studies that examined the impact of price, prescriptive and/or place policies on transitions, and for sub-question 2(c), we included studies that examined social or individual behavioural influences on transitions. We included quantitative measurements for all sub-questions, and planned to include qualitative evidence for sub-question 2(c) if relevant quantitative evidence was not identified.

For Review question 3, we included studies that reported on strategies used by the vaping industry in response to pricing, place, and/or prescriptive policies (described in Section 3.1), and effects of these strategies on vaping among the general population (with a focus on the following populations where the data exists: (i) young people (aged ≤25 years), (ii) people from different SES groups or (iii) people with mental health issues), worldwide. Any study type and publication type could be included.

Following de-duplication, records were imported into Rayyan for screening against selection criteria. Titles and abstracts were screened by one reviewer, with 10% checked by a second reviewer. All articles included at the abstract screening stage were examined at full text by one reviewer, with 10% checked by another reviewer ^17, 18^. Disagreements were resolved through discussion.

For review question 2a and 2c, we summarised the number of studies identified reporting on each of the transitions and each type of influence on behaviour. For review 2c we did not extract further information, but for all other review questions a data extraction sheet was developed. The data extracted, including study characteristics and information that could be useful for modelling, was tabulated in Section 7.2 and summarised narratively (reviews 2a, 2b and 3). Due to only one study being included for review 1, this was summarised narratively only.

Where UK evidence was missing, we briefly explored any key non-UK evidence that could be helpful for modelling, which was identified by the project team and with input from national and international stakeholders (see Section 7.4).

### 2.4 Conceptual modelling

Within the workshops we developed our understanding of the problem to be modelled by identifying stakeholders’ views of the key vaping behaviours that policy could affect, and which other behaviours interact with these vaping behaviours. We also voted on the priority policy themes, subgroups and outcomes of importance for modelling (See Section 3). We developed causal behavioural maps for each of the three priority policy themes, with each policy theme being considered by two groups within the workshop, leading to six initial maps. Within each theme the two maps were subsequently amalgamated into one map for each policy theme and verified within a project team meeting (see Section 4). We determined essential model requirements and desirable model requirements based on these workshop outputs. We used the outcomes of the workshop, the evidence identified from the reviews and the datasets and a modelling decision framework (the PHEM-B toolbox) ^19^ to provide recommendations for the types of modelling that could be constructed.

## 3. Priority research questions to be answered by decision-analytic modelling

### 3.1 Prioritising policy themes

Within six groups in our first workshop, stakeholders were asked to discuss and rank the below policy themes in order of their perceived importance (most [1st] to least [7th]) for decision-analytic modelling:

**Price**: To reduce access to harmful products by raising the retail price e.g., pricing of vapes.

**Place**: To reduce access to harmful products and encourage healthy alternatives by managing retailers and where consumption takes place e.g., changes to where people can vape or buy vapes.

**Promotion:** To inform people about the harms of consumption and promote healthy behaviours e.g., health-promotion messages targeting adolescents.

**Prescriptive**: To regulate the nature of harmful products and limit people’s exposure to marketing e.g., restrictions on marketing such as point of sale (POS) bans, plain packaging.

**Provider**: To limit the ability for industry to influence the formation and effectiveness of public policy, and to recoup the public costs generated by consumption e.g., regulation of the vaping industry.

**Product:** To restrict or put requirements on the products available e.g., banning of types of vapes or certain flavours.

**Person**: To strengthen the system of organisations and technology that encourages and supports people to quit or reduce consumption in the long term e.g., free provision of vapes to disadvantaged groups, support to quit vaping.

The policy types are based on a published paper about tobacco and alcohol policy conceptualisation ^20^, which drew on stakeholder input and the ‘P’s of marketing. The Tobacco and Vapes Bill was announced in the House of Commons two days before the workshop, so a summary of the proposed legislation was provided to all participants within the workshop. No pre-specified criteria were provided by which to choose policy themes so that this could be part of the group discussions when prioritising policy themes (for example, how modelling could help in relation to the Tobacco and Vapes Bill).

Stakeholders were grouped to ensure diversity within each group in terms of their expertise and geographical location. Each of the six groups were asked to rank the policy themes, with rationales. Following this, an initial round of anonymous voting with the entire stakeholder group was undertaken using online voting. The top three policies in this round with the highest ranking were Price, Prescriptive, and Product. Some stakeholders stated that they would have grouped the policy types differently and highlighted that the way in which industry uses these terms is different. The initial voting results were presented, and it was explained to stakeholders that we would focus on the top three policy themes from the second round of voting for the remainder of the workshop, after a whole group discussion of the initial outcomes. Within the whole group discussion, it was agreed that the policy theme “Prescriptive” captured policies under the “Product” theme (for example, banning disposable vapes is prescriptive) so we merged ‘Product’ and ‘Prescriptive’. A case was made for “Person” to be considered as a priority, given that targeting of vaping policy based on an individual's characteristics (such as socioeconomic position, or age) would be sensitive to the needs of particular groups of people who smoked or vaped. The importance of promotion was also highlighted since it was thought that education about the relative possible harms of vaping would be helpful.

**Figure 2.**
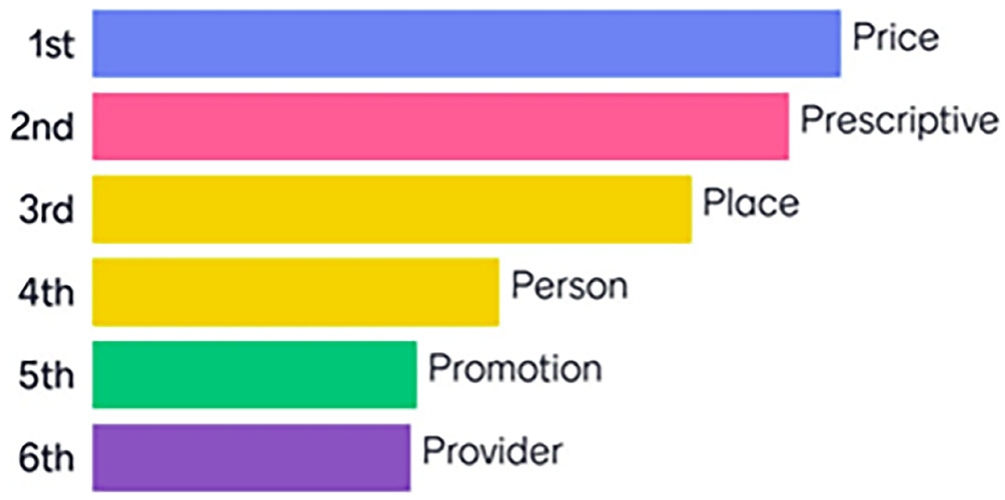
Summary of final policy theme ranking.

Following this a second round of voting took place and the top three policies with the highest ranking were Price, Prescriptive (including Product), and Place ( Table 1 and Figure 2). These were considered the priority policy themes throughout the remainder of this work.

**Table 1.** Distribution of final votes per policy (order of preference from most (1) to least (7) important).

Policies	1st	2nd	3rd	4th	5th	6th	7th
Price	24	9	1	3	0	3	0
Prescriptive	7	21	8	2	1	1	0
Place	5	7	18	5	0	4	1
Person	3	0	5	11	8	7	5
Promotion	0	1	2	6	13	12	3
Provider	1	1	4	3	11	8	10

## 4. Understanding the problem

To understand what is relevant to the problem of making decisions about vape policy, we developed systems maps of the problem, based on stakeholders’ advice. These provided a basis to prioritise variables to model, model type and systematic reviews of the empirical evidence.

### 4.1 Identifying smoking and vaping behaviours of interest

Within the workshop, groups identified which vaping behaviours (i.e., vaping initiation, regular vaping, vaping quit, vaping quit maintenance) are important for their assigned policy theme, which other behaviours interact substantially with these vaping behaviours, and what evidence exists about their interactions.

Across all themes (Price, Prescriptive and Place), all vape policies (e.g., increase in vape prices, marketing restrictions, retail restrictions) were thought likely to lead to behaviours that would result in a decline in vaping prevalence. Within the workshop, the majority suggested that policies that made vaping more difficult to access or less appealing could potentially lead to the following behaviours: declines in smoking quit attempts, and smoking quit maintenance, but increases in regular smoking (citing Friedman ^21^ and Khouja *et al.* ^22^), smoking uptake, and the use of other nicotine products (with potentially greater uptake in younger groups). It was also noted that although use of regulated vapes might decline, there might well be a rise in the use of unregulated (illicit) vaping products.

Some stakeholders, however, suggested that relationships between changes in vaping behaviour and smoking were unclear, including whether a decline in vaping would result in a change in smoking initiation in people who have never smoked, or changes in alcohol or illicit drug use. It was pointed out that the tobacco industry frequently raises concerns around illicit product use to stymie incoming regulation. Noting that all stakeholders were independent of industry, it was nonetheless felt important to consider empirical questions where there are little UK data available, including whether greater regulation of vapes will increase use of unregulated vaping products and tobacco smoking prevalence (through reduced quitting and increased uptake). Tobacco industry messaging is often employed to sow confusion among the public and policy stakeholders (see How big tobacco firms are using vapes to try to improve their image), requiring careful literature reviews in these areas as to inform understanding of the direction of key relationships.

### 4.2 Behavioural systems mapping

Within the workshop, groups were given a set of sticky notes with pre-written variables on each (as well as some blank ones to allow for additional variables) relating to:

- Inherent, sociodemographic and socioeconomic characteristics
- Lifestyle & psychological factors
- Macro- and meso-social structures
- Behaviours
- Behaviour-related harms
- Costs and other outcomes
- Government policy (not pre-populated)
- Vaping industry interventions (not pre-populated)

**Figure 3.**
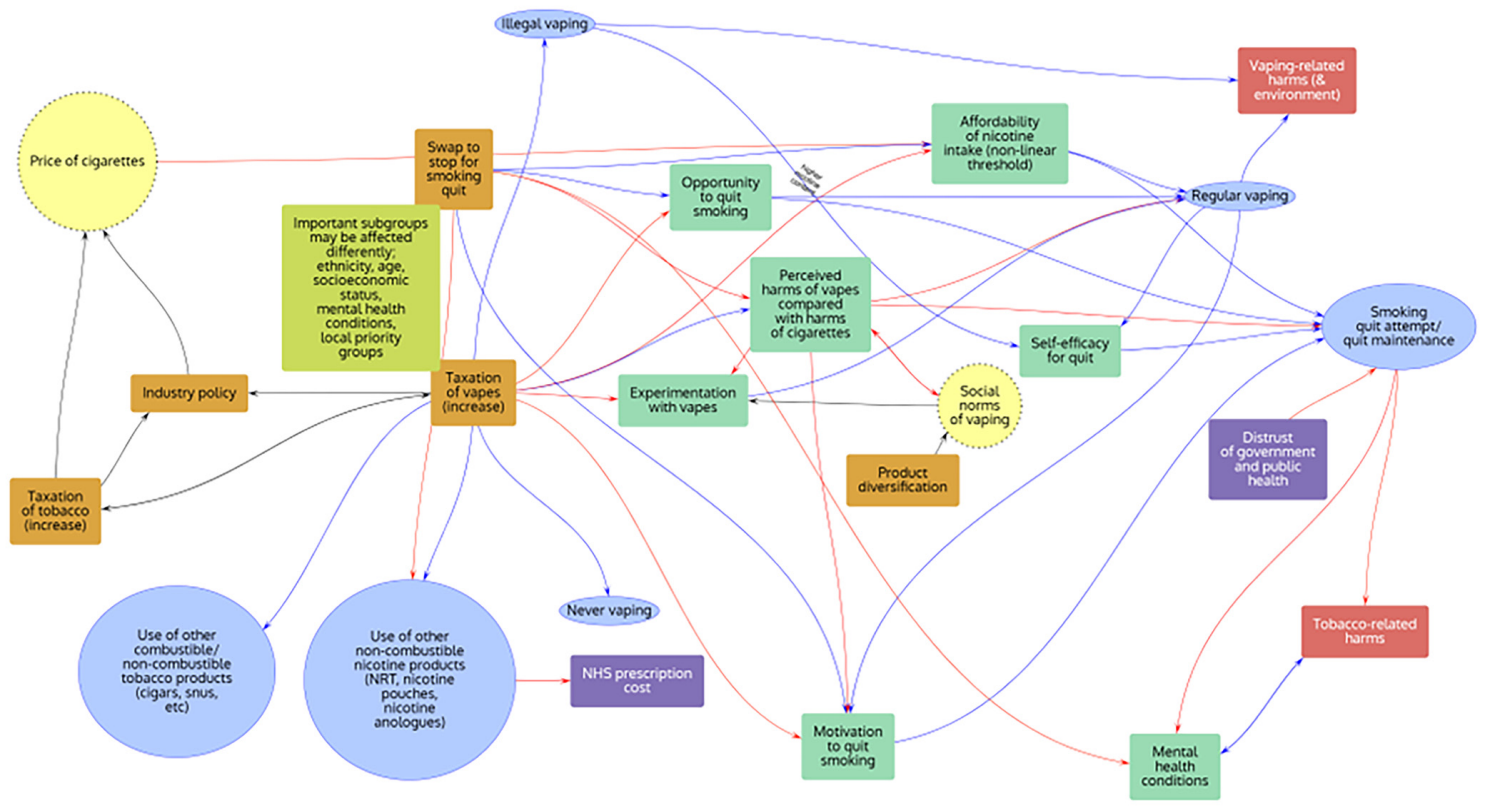
“Price” behavioural systems map. Note: Orange rectangles: Interventions; Yellow circles: Macro level; Green rectangles: Individual influences on behaviours; Blue ovals: Behaviours; Red rectangles: Harms; Purple rectangles: Other outcomes.

Groups were asked to choose the most important factors influencing the behaviours of interest identified within the workshop based upon their expertise, and to draw arrows between factors to show how each factor might affect the other included factors. The resulting behavioural systems maps for each policy theme were subsequently digitised using PRSM ^23^ and are shown in Figure 3– Figure 5 below.

**Figure 4.**
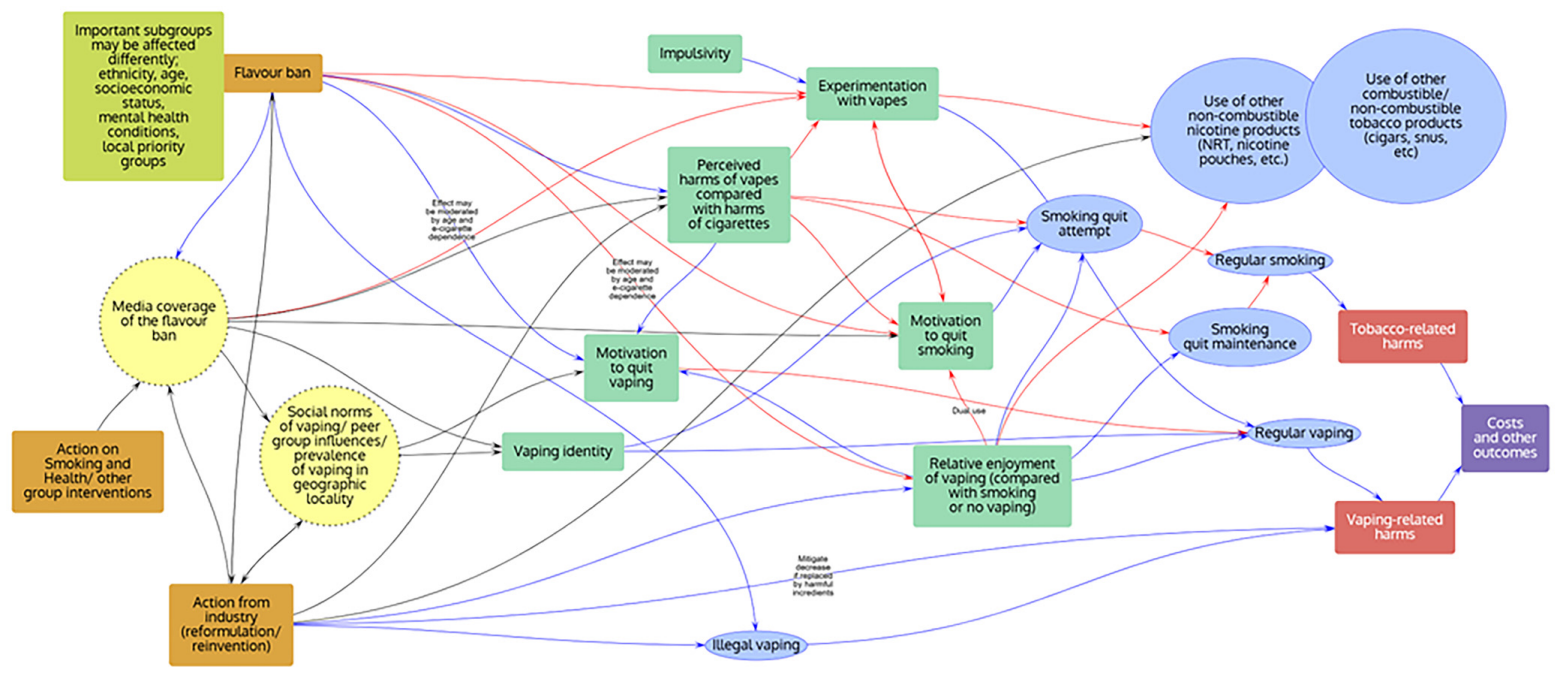
“Prescriptive” behavioural systems map. Note: Orange rectangles: Interventions; Yellow circles: Macro level; Green rectangles: Individual influences on behaviours; Blue ovals: Behaviours; Red rectangles: Harms; Purple rectangles: Other outcomes.

**Figure 5.**
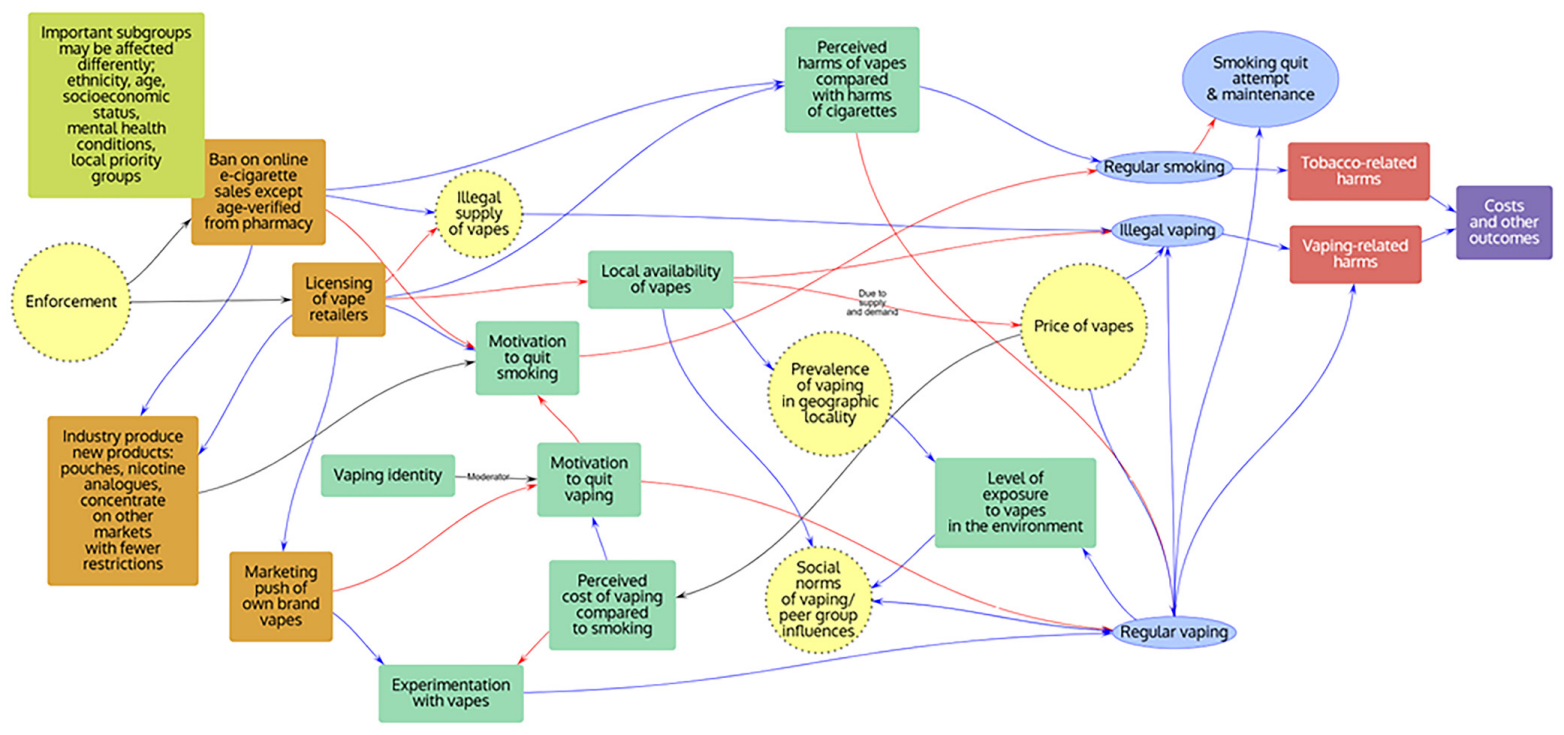
“Place” behavioural systems map. Note: Orange rectangles: Interventions; Yellow circles: Macro level; Green rectangles: Individual influences on behaviours; Blue ovals: Behaviours; Red rectangles: Harms; Purple rectangles: Other outcomes.

Across all policy themes, the following factors were considered to be important by stakeholders:

- age;
- socioeconomic status;
- perceived harms of vapes compared to cigarettes;
- experimentation with vapes;
- vape dependence;
- motivation to quit smoking;
- social norms;
- regular vaping;
- regular smoking;
- smoking quit attempt;
- smoking quit maintenance;
- use of other non-combustible nicotine products;
- illicit vaping;
- tobacco-related harms;
- vaping-related harms.

***Policy Theme 1: “Price” (Groups 1 and 2; Figure 3)***

The theme highlighted the dual importance of regulation to increase the price of vapes (e.g. through the proposed e-liquid duty) to reduce vaping where it is not acting to reduce tobacco use, and of providing free vape starter kits (e.g. through the Swap to Stop scheme) as a way of promoting and increasing the accessibility of vaping to people who would use them to reduce tobacco use but who may be price sensitive ^7^. There were no factors that were considered to be important across both “Price” theme maps beyond those already considered important across all themes. However, the macro-level variables “price of cigarettes” and “price of vapes” were highlighted as important within Group 1. After the workshop, one participant also highlighted that the perceived costs of vaping relative to smoking may be important, which is consistent with the government’s rationale for proposing a one-off increase in tobacco duty alongside their proposed new duty on vape e-liquids. In the case of cigarette pricing, the tobacco industry has been shown to have under shifted taxes on the lowest priced brands and market small pack sizes ^24, 25^.

***Policy theme 2: “Prescriptive” (including “Product”; Groups 3 and 4; Figure 4)***

In addition to those factors considered to be important across all themes, relative enjoyment of vaping (compared with smoking or not vaping) and motivation to quit vaping were included in both “Prescriptive” theme maps, e.g. as influenced by flavour bans or bans on disposable vapes. Media coverage, e.g. of the health and environmental harms of vaping and policy to address these, was also considered to be an important factor across “Prescriptive” theme groups, both influencing micro- and macro-level variables, with media coverage being influenced by government policy, industry and non-government groups such as ASH.

***Policy Theme 3: “Place” (Groups 5 and 6; Figure 5)***

In addition to those factors considered to be important across all themes, perceived cost and local availability of vapes, e.g. as may be influenced by retail licensing regulations, were thought to be important factors influencing behaviour across both “Place” theme maps. This theme also recognised the importance of the opposing actions from industry to government policy which could impact individual behaviour, e.g. through product innovation and targeted distribution. After the workshop, one participant also highlighted that it may be interesting to test the impact of the proposed extension of vape free places on vaping prevalence and how it interacts with the other policy themes.

## 5. Potentially useful modelling approaches

### 5.1 Useful modelling approaches according to existing taxonomies

The choice of modelling approach is dependent on the characteristics of the problem and the decision makers’ requirements, as well as on what is feasible. We have developed an understanding of the problem in Section 4. The goal of this work was to undertake data mapping for a model of vape policy which would be useful to a range of decision makers. This may mean that different modelling approaches would be useful for different decision makers and/ or be feasible in different decision-making contexts. We therefore recommend setting up a platform to flexibly and coherently assess policy options (see Section 8.3.10).

When policy makers would like to understand the impacts of interventions upon people with diverse characteristics and/or histories, and/ or when interactions between individuals can affect outcomes, as is generally the case for vaping, an individual-level model rather than a cohort approach should be considered ^9^. This means that multiple individual characteristics such as age and current or past smoking or vaping status can be incorporated, and these characteristics can affect what happens to the individual in the model. These models can also be extended to include theory-informed factors and mechanisms (e.g. how vaping identity, as a motivation, influences behaviour) and complexity-informed perspectives that incorporate feedback loops and interactions amongst model elements to capture emergent outcomes. The guidance on the use of such complex systems models by Breeze *et al.* ^26^ suggests that a complex system model would be preferable for vape policy modelling because the outcomes of the interventions are dependent on many interacting factors, including other people’s smoking and vaping behaviours and the social and industry context. This is true for all the key priority policy themes: price, place and prescriptive policies. However, as emphasised by our international stakeholders, a balance must be struck between model complexity versus the understandability and timeliness of model evidence. One of the benefits of following a systematic process to model development through understanding the priority questions and available data, as we have done in this work, is that it can support thinking about the appropriate level of model complexity.

We used a toolbox developed to help modellers incorporate the influences on behaviour into public health economic models (the PHEM-B toolbox) to identify which approaches might be useful ^19^. Methods that would ideally be used, given the complex system within which the policies are being evaluated, are:

a) Collaboration between modellers and behavioural/ social scientists throughout.
b) Reviewing the literature for the behavioural theories used to develop the intervention(s) to understand the problem in behavioural terms and identify the influences on behaviour.
c) Applying a behaviour change intervention ontology ^27^.
d) Behavioural systems mapping (see Figure 3– Figure 5).
e) Econometric analyses to estimate statistically the relationship between smoking and vaping behaviours and pricing changes or prescriptive changes to vaping product characteristics. These could be analyses of existing datasets (e.g., 28) or of behavioural experimental studies (e.g., 29).
f) Agent-based modelling to describe the influences on behaviours, the interactions between individuals and their access to place (e.g., 30)
g) Social network analysis to model the interactions between the vaping and smoking behaviours of individuals (e.g., 31)
h) Spatial analysis to model place-based vaping policies (e.g., 32).
i) Theory-informed statistical analysis will be needed to quantify behavioural theory linking the influences on smoking and vaping to vaping and smoking behaviours (e.g. 33)

For more information about each of these methods, see Squires *et al.* ^19^

However, such a research programme would require substantial resources. It may be more feasible in the short term for modellers and behavioural scientists to collaborate to develop version 1 of a “core” individual-level model of vaping and smoking behaviours. Additional complexity can then be developed over the medium to long term, expanding the range of modelling approaches, model complexity, and the population subgroups to which models are tailored. In addition to time and resources, the choice of modelling approaches is dependent on data availability, accessibility of any existing relevant good quality models and expertise of the modellers ^9^. After considering existing modelling studies in Section 5.2, the model scope and requirements in Section 6 and reviewing the existing evidence in Section 7, we will provide short-term and longer-term recommendations about feasible modelling approaches and data requirements for vape policy modelling in Section 8.

### 5.2 Existing modelling studies

There are two recently published systematic reviews of modelling studies for vape policies ^10, 11^, including 32 studies between them.

Most models used US data and only two have been applied to the UK context. Levy *et al.* ^13^ used the SimSmoke model which has been applied to several countries. An ‘indirect simulation method’ was employed that did not involve explicitly modelling vaping and dynamics. Instead, the SimSmoke model predicted post-2012 smoking prevalence based on pre-2012 data which was considered to be a no-vape counterfactual. The impact of vapes was estimated by comparing data on smoking trends post-2012 from survey data with those predicted by the model. This analysis suggested that the use of vapes helped to reduce smoking prevalence from 2012–2019.

Kalkhoran *et al.* ^12^ employed a decision-tree design to estimate the impact of various vape promotion interventions from a UK and US perspective. The decision tree included population-level transition probabilities from an initial state (no cigarette, no vaping) to one of 5 final states: never use of cigarettes or vapes, cigarette use, vaping, dual use of cigarettes and vapes, or cigarette quit. Scenarios which were tested where vapes were only used by people who smoke or those with a propensity to smoke, led to population level benefits of vaping, whilst those where vapes were taken up by youth who would have never smoked showed net health harms of vaping.

Data from both the UK and US were used for parameterisation for these two UK models. Key UK data sources from these two models included: UK Office for National Statistics (ONS) data; the Smoking Toolkit Study data for England; Action on Smoking and Health data; Scottish Schools Adolescent Lifestyle and Substance Use Survey; a cross-sectional survey of year 6 (10–11-year-old) children in Wales; and the International Tobacco Control (ITC) Four Country Survey. Neither of these models would allow the analysis of the impact of vape policy on important subgroups of the UK population.

The majority of the studies included in the two systematic reviews are cohort Markov models, with only six individual-level studies that reported population level outcomes ^34– 39^. Given that we identified that an individual-level model would be preferable for vape policy modelling in Section 5.1, we consider these six studies in further detail here. There were two individual-level dynamic simulations ^34, 35^ which consisted of three smoking states; never smoker, smoker and former smoker. Vaping was not included within the states but impacted the probability of moving between never smoker and smoker and smoker and former smoker. National Health Interview Survey (NHIS) data was used to inform the baseline smoking transitions in the models. The impact of vaping on the smoking initiation rate was based on a study by Soneji *et al.* ^40^, whilst the impact of vaping on the smoking cessation rate was based on studies by Beard *et al.* ^41^, West *et al.* ^42^, and Zhu *et al.* ^43^ and these were not age-related. The inclusion of any harms of vaping was limited in these studies.

There were four agent-based models which did include states for both tobacco smoking and vaping, and some varied by age ^36– 39^. The data sources used for calibrating the transitions between behavioural states included the National Health Interview Survey (NHIS), Population Assessment of Tobacco and Health (PATH), ADJUSST (Adult JUUL Switching and Smoking Trajectories), US Census, the National Youth Tobacco Survey (NYTS), US Surgeon General and Centre for Disease and Control Prevention (CDC) data. Two of the agent-based models incorporated the impact of a social network upon vaping; however, the data upon which these were based were very limited.

The results of the existing vape policy models were mixed in terms of whether vape policies resulted in net population health harms or benefits. Key drivers of outcomes across the models were:

1. Relative safety of vaping compared with smoking and compared with no smoking or vaping;
2. Smoking cessation rate via vaping;
3. Assumed reduction in health consequences associated with being a person who formerly smoked compared with being a person who currently smokes;
4. Transition into smoking from vapes among people who have never smoked;
5. Age of people in the model. Net harms were more often predicted if models included younger people who could vape in the model (age 12+, 15+);
6. Inclusion of states for dual use or allowing for people who previously smoked to take up vapes.

It will therefore be important to identify good evidence around the parameters and assumptions relating to key drivers 1–4 and to quantify uncertainty around the estimates. With regards to key driver 5, it will also be essential for any vape policy model development process to understand the implications of the model population included within the models, particularly in terms of age, where excluding younger age groups could underestimate the harmful effects of vapes, as would omissions of certain states (key driver 6). In addition, the choice of model structure and behavioural states included could have a substantial impact on model results. Given the current dearth of UK models of vape policy, a *de novo* model is required, which is flexible and open source.

## 6. Model scope and requirements

### 6.1 Policy-relevant outcomes and subgroups

**Figure 6.**
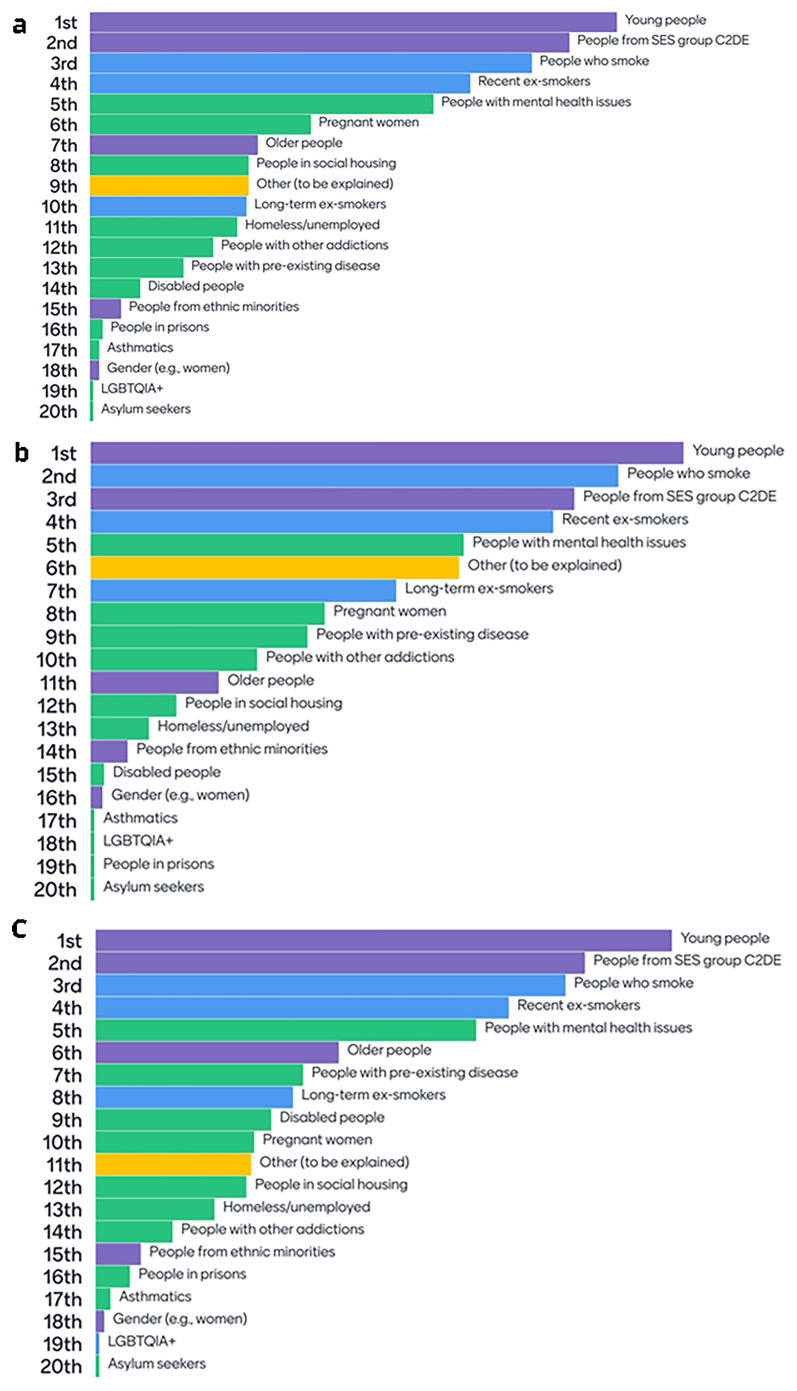
**a**. Subgroup rankings related to “Price” policies. **b**. Subgroup rankings related to “Prescriptive” policies. **c**. Subgroup rankings related to “Place” policies. Key: Blue = Vaping and smoking subgroups; Purple = Sociodemographic groups; Green = Priority health groups; Yellow = Other (specified).

**Figure 7.**
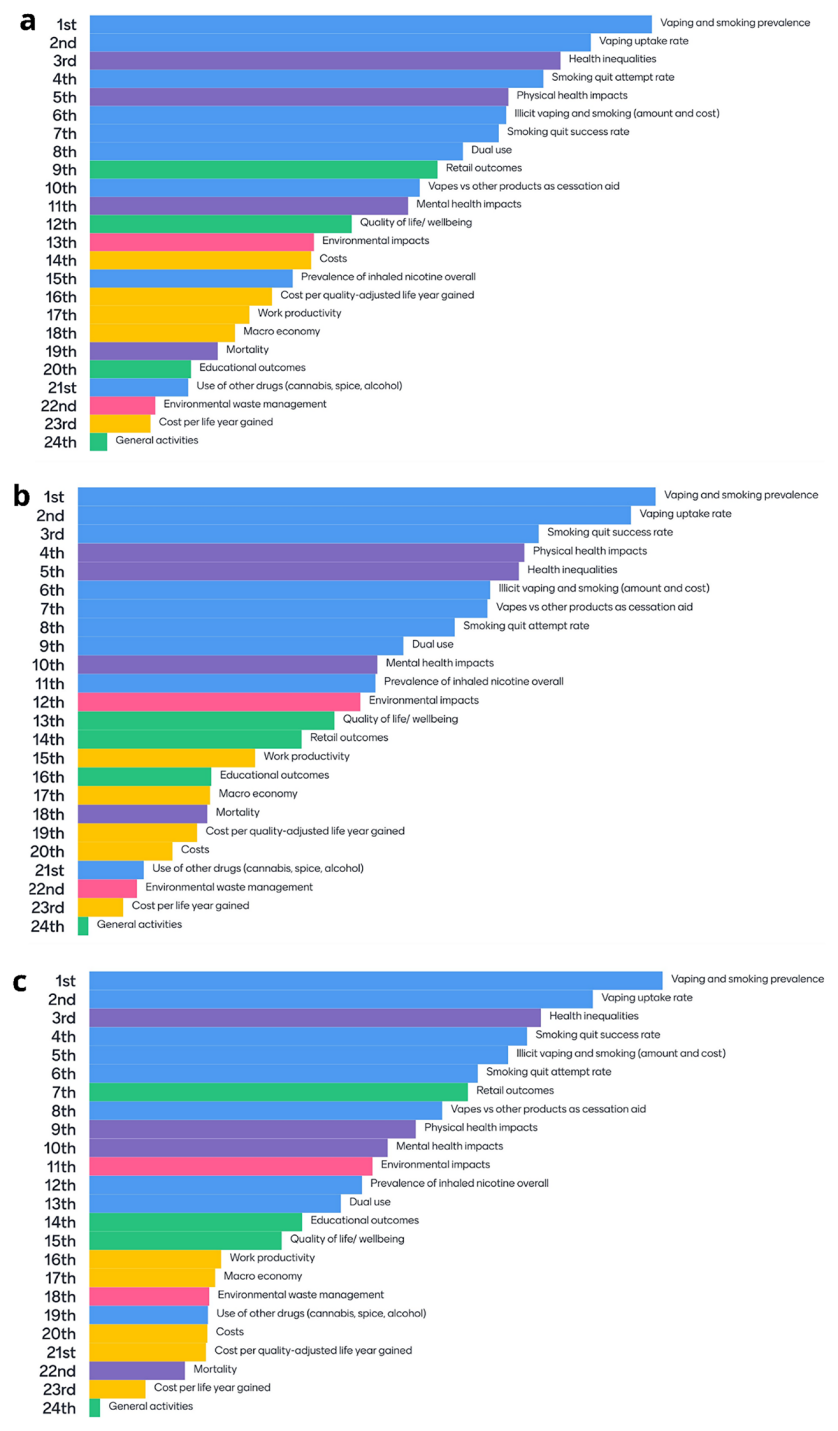
**a**. Outcome rankings related to “Price” policies. **b**. Outcome rankings related to “Prescriptive” policies. **c**. Outcome rankings related to “Place” policies. Key: Blue = Vaping, smoking and nicotine use outcomes; Purple = Health outcomes; Green = Other outcomes; Yellow = Economic outcomes; Pink = Environmental outcome.

At the first stakeholder workshop, stakeholders were asked to consider a list of policy-relevant subgroups and outcomes that should be considered in decision analytic modelling and to rank them in order of importance. Following two rounds of discussion and voting, there were similarities in the prioritisation of subgroups and outcomes across all three policy themes. The top three highest ranked subgroups were young people, people experiencing socioeconomic disadvantage (in this case operationalised according to occupational grade [C2DE includes skilled manual workers, semi-skilled and unskilled workers, and those relying on state benefits or unemployment]), and people who smoke. Recent ex-smokers were ranked the fourth most important subgroup across all policy themes, followed by people with mental health conditions ( Figure 6a–c). The top 10 outcomes prioritised by stakeholders were dominated by vaping, smoking and nicotine use outcomes. Other outcomes ranked in the top 10 included “Health inequalities”, “Physical health impacts” (discussed as including “Mortality”), “Retail outcomes” (convenience store footfall) and “Mental health impacts” ( Figure 7a–c).

**Figure 8.**
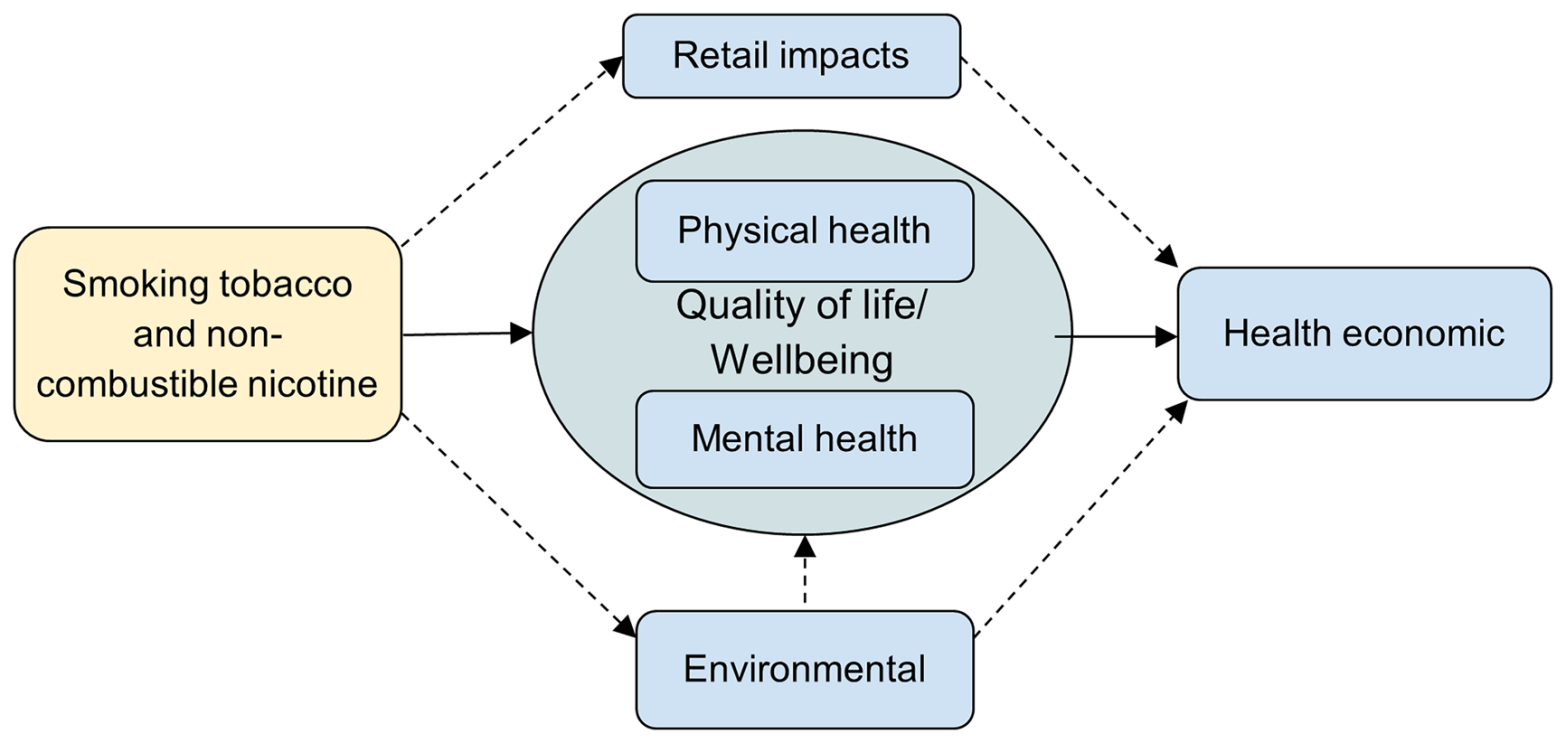
The relationships between important outcomes.

It was also noted that some of the outcomes could be on the pathway to other outcomes (e.g., smoking tobacco and non-combustible nicotine use could impact on environmental outcomes which could impact on physical and mental health). We have depicted the relationships between the outcomes in Figure 8. Following the workshop, the project team discussed that while in theory all the outcomes could feed into a health economic analysis, within this project we focus on the relationships depicted by the solid arrows only. Future research could consider evidence from other fields to inform the relationships denoted by the dotted arrows.

### 6.2 Which behaviours should be included in a model?

Within the workshop, stakeholders highlighted many behaviours that could be affected by vaping policies, directly or indirectly. However, a model would become extremely complex and difficult to parameterise if too many behaviours were included. Therefore, based on the behavioural grid developed in the workshop considering the key interactions with vaping behaviours and the behavioural systems mapping exercise, the most important behaviours that would substantially influence or be influenced by vape policies and therefore should be included in any vape policy model are:

A. Regular vaping
B. Regular tobacco smoking
C. Tobacco smoking quit attempts and maintenance of the quit

Use of other non-combustible nicotine replacement products such as nicotine pouches or licensed nicotine replacement therapy could also impact vaping and so should be included in a model if feasible.

Illicit vaping (use of unregulated vapes), while considered to be an important interacting behaviour with vaping, would be difficult to model due to the difficulties in obtaining this data. There is currently no established approach for measuring illicit vaping or availability, and often from the consumers’ perspective unregulated vapes look the same as regulated vapes and they may not be aware that they are using an unregulated vape. Nonetheless, models evaluating policies around trading standards for vapes, in particular, would ideally include illicit vaping.

Within this first workshop, less importance was placed on quitting vapes. Whilst some people highlighted that the use of other conventional and newer nicotine and tobacco products, e.g. nicotine pouches, could interact with vaping, this relationship was not often included in the behavioural systems maps as the behaviours listed above.

### 6.3 Essential and desirable data requirements for a vape policy model

Within a model we need to describe the impact of vaping policies upon vaping and smoking behaviours over time, and to describe the short- and longer-term impacts of those behaviours upon relevant outcomes. As described in Section 5.2, there has been limited vape policy modelling in the UK context, and as such a *de novo* model will be required. There is therefore a need to describe the minimal requirements for a first working version of a model that can help to inform policy decisions in the shorter term, with more desirable modules to be added over the longer term. These requirements are based on the first workshop outcomes and the use of the PHEM-B toolbox to identify potentially useful modelling approaches (see Section 5.1). The completed decision framework from the toolbox is available at https://osf.io/8zaxc/.

It is essential to identify evidence to model:

1) the transitions between vaping and smoking and the interactions between them over the lifetime of a group of heterogeneous individuals, including changes in prevalence of smoking and vaping over time (important individual characteristics include age, SES and people with mental health conditions);
2) how price, place and prescriptive policies will affect vaping and smoking behaviours;
3) the relative health harms associated with vaping compared with smoking and the health harms of vaping compared to never smoking or vaping.

It would be desirable to have data to model:

4) the influences on behaviour and the mechanisms of action of policies affecting vaping and smoking behaviours, including the interactions between socioeconomic factors, psychological factors, social networks, spatial factors and institutional, structural and cultural variables (e.g., individual perceptions of the relative harms of smoking versus vaping which may be influenced by social networks and the media);
5) cross price elasticities of demand between vaping and tobacco products (for “price” policies), and the influence of prescriptive policies for vapes, e.g. flavour bans, on the use of tobacco products;
6) the potential impact on vaping and smoking behaviours of ways in which industry might try to mitigate the effects of government policy;
7) the additional harms of use of regulated (licit) over unregulated (illicit) vapes;
8) the interaction between vaping and use of other products for smoking cessation;
9) smoking and vaping dual use outcomes;
10) environmental outcomes associated with vapes;
11) retail outcomes associated with vapes;
12) the costs and quality-adjusted life years (QALYs) associated with new policies.

## 7. Current evidence

Extensive reviews have already been undertaken in the UK context on the health harms of vapes compared with smoking tobacco and on the use of vapes as a smoking cessation aid. We briefly summarise this evidence in Section 7.1 before presenting our reviews which were designed to fill current evidence gaps in Section 7.2. Section 7.3 then sets out UK datasets which could be useful for modelling vape policies. Finally, Section 7.4 briefly summarises non-UK data where limited UK data has been identified and non-UK data could be potentially useful for modelling.

### 7.1 Summary of key existing evidence reviews

***7.1.1 Standard vaping products***

 Key systematic reviews (commissioned by DHSC and Cancer Research UK, on behalf of the Royal College of Physicians), of studies of the health effects of vapes conducted in UK and international settings ^1, 7^, include a synthesis of studies of biomarkers of exposure (BoE) and potential harm/effect (BoPH). BoE indicate uptake of specific toxicants, while BoPH, such as lung function or inflammation, serve as early indicators of disease before clinical outcomes emerge. These two reviews identified 261 (human) studies published between August 2017 and February 2023 comparing people who vape, people who smoke, people who do both (dual use), and people who do neither (non-use).

Across the two reviews, two studies by Shahab *et al.* ^44^ and Richmond *et al.* ^45^ were conducted in the UK, the latter of which included people who had never smoked. Very few studies conducted in other countries included people who never smoked (defined as smoking fewer than 100 cigarettes in their lifetime). Both reviews reported that levels of nicotine and its metabolites in people who vape are similar to or lower than those who smoke. The reviews also highlight that nicotine concentration in vapes drives intensity of puffing (compensatory use) in order to achieve preferred nicotine levels. Thus lower nicotine strength vapes result in more intense puffing and thus likely greater exposure to BoE and BoPH (e.g., formaldehyde). Generally, levels of tobacco-specific nitrosamines, volatile organic compounds and polycyclic-aromatic hydrocarbons were lower in people who vaped than in those who smoked and were higher or similar to people who did neither (i.e. people who formerly or never smoked or vaped).

Both articles concluded vaping exposes people who vape to a much narrower range of toxicants than smoking, levels of toxins absorbed from vaping are generally low and therefore it is likely that vaping poses a small fraction of the risk of smoking ^1, 4^. Both reviews also recommended people who smoke should be encouraged to use vaping products (or medicinally licensed products) for stopping smoking or as alternative nicotine delivery devices to reduce the health harms of smoking. By contrast, people who had never smoked or had formerly smoked should be discouraged from taking up vaping (unless the person would otherwise relapse to smoking) as the degree of long-term residual absolute risk from vaping compared with non-use of tobacco or nicotine products remained unclear, but not negligible ^1^.

Studies included in both reviews also had several limitations including inconsistent definitions of vaping, smoking and dual use status. The latter incorporates very heterogeneous behaviours often not properly accounted for in studies ^46– 48^. For instance, daily use of vapes with non-daily cigarette smoking does reduce exposure, but daily use of cigarettes with non-daily vaping does not ^49^. Dual use patterns therefore likely determines exposure to harmful constituents. Typical patterns, characterised by predominant smoking, are therefore less likely to carry health benefits ^50^; Non-users were also poorly defined. In addition, there was a lack of consideration for previous smoking history, socioeconomic status and environmental exposure. Methodological heterogeneity in measurement meant only a handful of studies could be meta-analysed. Most of the current research on biomarkers is related to BoE, and it is not clear if low levels of exposure translate into improved clinical outcomes and averted smoking-related diseases. There is a limited (but growing) number of studies examining BoPH or health impacts in individuals with pre-existing conditions, and other key systematic reviews (identified by international experts) broadly agree that there is insufficient high-quality data about the absolute and relative long term health effects of vaping ^51^.

Vapes are an effective smoking cessation aid. Cochrane reviews of trials comparing nicotine-containing vapes with placebo (nicotine-free) vapes, with behavioural support or nicotine replacement therapy (NRT) estimate that vapes increase 6–12 months abstinence rates by between 46 and 88% ^2^. A recent Cochrane network meta-analysis concluded that vapes, together with varenicline and cytisinicline (cytisine), are among the most effective smoking cessation aids ^52^. Similarly, observational studies of the real-world effectiveness of vapes show these to be among the most effective smoking cessation aids in the UK ^3^. Their effectiveness has been demonstrated across population subgroups, including people that smoke across different ages and socioeconomic positions, and those with mental health conditions ^3, 7, 53– 55^. They are also the most popular: in 2023/24, they were used in 40% of attempts to quit smoking in England – more than twice as many as the next most popular aid (NRT bought over-the-counter, used in 17%) ^3^. However, many of those who quit smoking with vapes continue vaping long-term ^56^, which could have financial and (mental) health implications ^57^.

***7.1.2 Illegal vaping products***

Illegal vaping products are products that do not meet the requirements of the Tobacco and Related Products Regulations or the General Products Standards Regulations. This includes products which do not comply with regulations such as labelling or limits on nicotine concentration; products which contain illegal ingredients, which may include banned (e.g., diacetyl) or illicit substances (e.g., Class A drugs) in addition to or instead of nicotine; and products which have not been registered with the MHRA. Illicit substances may also be added to legal products after purchase through ‘DIY mixing’. Further, and not related to the vaping product itself, vapes may be sold illegally to children/under 18-year-olds (irrespective of whether they are legal or illegal products).

There is limited evidence directly comparing the impact of use of illegal vapes. Nonetheless, it is likely that these products carry a bigger risk than legal vapes, given the lack of oversight and regulation of what they contain. This is particularly true for vapes that contain banned/illicit substances. For instance, an outbreak of acute lung injuries and a number of deaths in the US was caused by the sale of illicit tetrahydrocannabinol (THC) vapes contaminated with vitamin E-acetate in the US ^58^, and adverse events have been reported by users of vapes contaminated with illicit synthetic cannabinoids in the UK ^59^. This appears to be a growing problem as seizures of non-compliant nicotine vapes, vapes containing illicit drugs and of vapes sold illegally to under-18s have grown by 59% in 2023/24 ^60^. These issues are interlinked insofar as vapes containing illicit drugs are also sold to underage vapers as evidenced by a recent study of vapes seized from 27 English secondary schools in 2023/24, which contained synthetic cannabinoids, often purchased by pupils as cannabis vapes. These were most commonly found in refillable devices and were more prevalent in areas of higher deprivation, raising concerns about youth exposure to both illegal vapes and illicit drugs ^61^. However, even the illegal sale of legal vaping products to minors is a concern, as animal studies have shown that nicotine affects the developing adolescent brain differently to the adult brain, potentially resulting in behavioural, emotional and cognitive dysregulation and greater levels of addiction ^62^. The long-term health risks of vaping are currently unknown, but continued vaping is unlikely to be risk-free.

### 7.2 Results of literature reviews

***7.2.1 Included studies***

**Figure 9.**
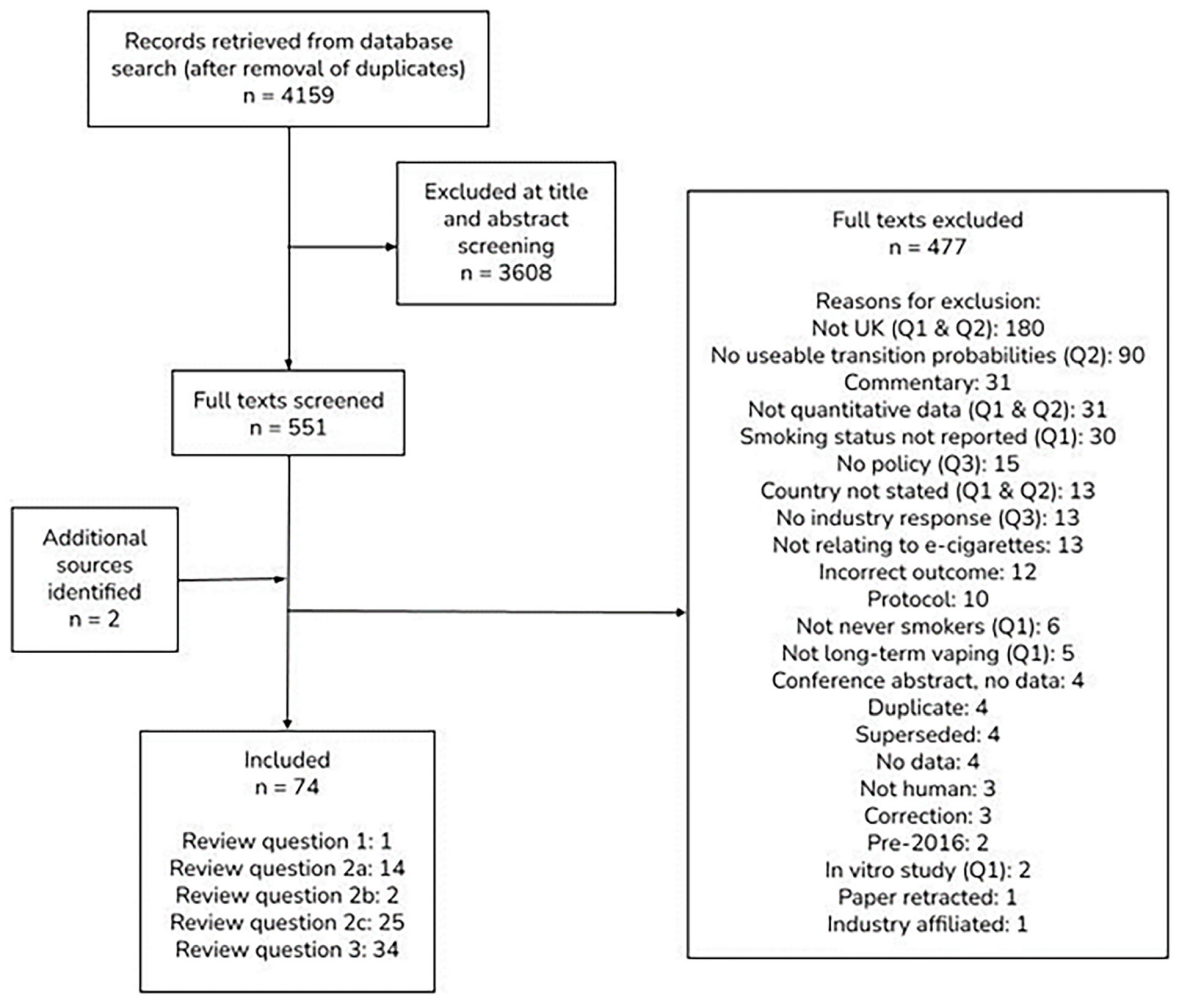
PRISMA diagram.

 Searches yielded 4,159 records following de-duplication, and one additional source was identified, and 3,609 were excluded at title and abstract screening. Full texts for the remaining 551 records were examined. Reviews of evidence were found to be predominantly non-UK studies and hence the decision was made to review the primary studies only. A total of 77 studies were included in the review; one in Review Question 1, 14 in Review Question 2a, two in Review Question 2b, 25 in Review Question 2c and 34 in Review Question 3, as shown in Figure 9.

***7.2.2 Review question 1: UK evidence on health and wellbeing impacts***

There was only one UK study reporting on the health and wellbeing impacts of vapes on people who have never smoked (N=117). This was a prospective cohort study that examined the impact of vape and cigarette consumption on the DNA methylation profile associated with lung cancer ^45^. The study population was young (mean age 21) with relatively short vaping duration and limited lifetime smoking exposure. The authors found that the methylation profile for vaping is distinct from, and far less pronounced than, the cigarette smoking profile and (unlike for smokers) was not informative for distinguishing carcinoma from adjacent normal tissue. Two studies, published after the rapid reviews’ search end date identified through stakeholder input, report findings from a sample of 364 adolescents (mean age 17.6) in England, the US, and Canada. In the first ^6^, nicotine exposure was similar among those who exclusively vaped, exclusively smoked, or used both in the past 7 days, and significantly higher than among non-users. Among vapers, nicotine exposure did not differ by product strength (>20 mg/mL vs ≤20 mg/mL) but was higher among those using nicotine salt products compared to non-salt or “don’t know” users.

In the second ^63^, exclusive vapers had lower exposure than smokers and dual users to most volatile organic compounds (VOCs) tested, including acrolein, acrylamide, and acrylonitrile. However, exposure to toluene was higher among exclusive vapers than dual users and non-users. In a sensitivity analysis, acrylamide levels were also higher among adolescents who had vaped in the past 24 hours compared with those who had not smoked or vaped.

Given the lack of UK evidence for this review we describe some of the non-UK evidence on this topic in Section 7.4.

***7.2.3 Review question 2: Transition probabilities between smoking and vaping***

We identified 14 UK studies which examined transition probabilities between smoking and vaping states, two studies which reported the impact of policies on transition probabilities and 25 studies which quantitatively reported the impact of social, structural or individual behavioural influences on the transitions between vaping behaviours.

**Transition probabilities between smoking and vaping states in the UK**

**Figure 10.**
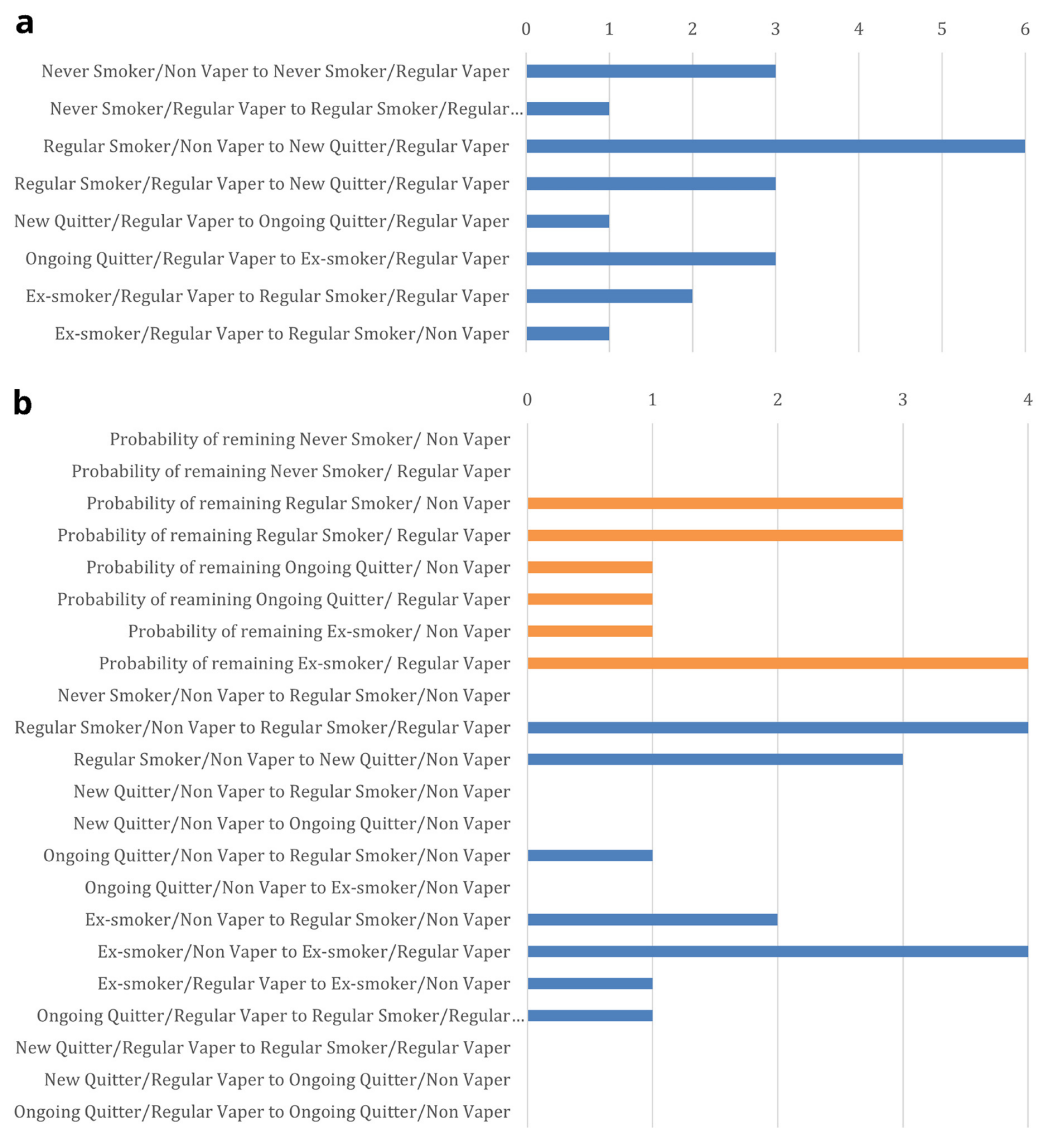
**a**. Number of studies for each priority transition. **b**. Number of studies for each other transition. Note: Orange bars are the number of studies which report the probability of remaining in a behavioural state; blue bars are the number of studies which report transitions to different behavioural states.

Figure 10 shows the number of UK studies identified for each transition between smoking and vaping. Based on the outcomes of the first workshop, smoking behaviours of interest were divided into people that have never smoked ‘never smokers’, people that smoke ‘current smokers’, people that have quit smoking in the last month ‘smoking quitters’, people that quit smoking between 1 and 12 months ago ‘ongoing quitters’ and people that quit smoking more than 12 months ago ‘ex-smokers’. In order to reduce possible permutations and given the current lack of evidence around latent vaping harms following vaping quit and the findings that vaping harms are dominated by smoking harms, we reported vaping only in terms of ‘vaper’ or ‘non-vaper’.

For full data extraction, we focused on those studies which reported priority transitions for vape policy modelling, as agreed by the project team, set out in column 1 of Table 2. Relevant evidence was found for each priority transition.

**Table 2.** Studies reporting transition probabilities relating to both smoking and vaping behaviours.

Transition	Author (year)	Data/Setting	Qualitative description of how the quantitative estimates are estimated	Result	Significance/CIs
Never Smoker/Non Vaper to Never Smoker/Regular Vaper	Moore *et al.* (2020) ^69^	School-based surveys (SHRN/HBSC Wales, SDDU England)	Odds Ratios (OR) from logistic/segmented logistic regression for 2016/2017 vs 2014/2015	1.55 (2014 to 2016)	P=0.074 95% CI (0.96 – 2,52)
				1.14 (2015 to 2017)	P=0.083 95% CI (0.98 -1.31)
	Jackson *et al.* (2024) ^70^	Adults (18+) surveyed as part of the Smoking Toolkit Study in England	Prevalence percentages (%) with 95% CIs; Trends modelled using logistic regression for how many ‘never smokers’ now use vapes	Prevalence of 3%	95% CI (2.3% to 3.8%)
	Parnham *et al.* (2024) ^66^	10–25 year olds in the UK Household Longitudinal Study (data from 2015-2021) with at least 2 waves of data.	Probability of transitioning based on a continuous time multistate Markov model after 1 year.	4.0%	95% CI (3.7% to 4.2%)
Never Smoker/Regular Vaper to Regular Smoker/Regular Vaper	Parnham *et al.* (2024) ^66^	10–25 year olds in the UK Household Longitudinal Study (data from 2015-2021) with at least 2 waves of data.	Probability of transitioning (from never smoker, regular vaper to regular smoker, with any vaping status) based on a continuous time multistate Markov model after 1 year.	14.3%	95% CI (12.7% to 16.2%)
Regular Smoker/Non Vaper to Smoking Quitter/Regular Vaper	Simonavicius *et al.* (2020) ^64^	Online survey of UK panel members (Ipsos MORI). Heavy smokers.	Used latent transition analysis to estimate probability of moving between classes for a 16 month period	3.1%	Not given
	Jackson *et al.* (2024) ^70^	Smoking Toolkit Study	Infers patterns of uptake/transitions by examining trends in vaping prevalence among people who have different lengths of smoking quit. Prevalence as of 2024.	Prevalence of 41.4% among people using e-cigs to quit smoking	95% CI (37.7% to 45.2%)
				Prevalence of 13.7% among people who stopped smoking completely in the past year who did not use e-cigs to quit, who are now using e-cigs	95% CI (9.7% to 19.0%)
	Jackson *et al.* (2024) ^70^	Smoking Toolkit Study	Adjusted Odds Ratios (ORadj) with 95% CIs; Bayes Factors (BFs) of a smoker becoming a quitter if they use vapes versus not using vapes	1.95 (18yo to 64yo)	Sig (<0.05) 95% CI (1.72 – 2.21)
				1.5 (65+)	NS (p~0.07) 95% CI (0.96 – 2.34)
	Hajek *et al.* (2019) ^65^	Adults that smoke (18+) seeking help to quit via StopSmokingService, not currently using vapes	Abstinence rates (%); Risk Ratio (RR) of quitter using an vape starter quit compared to people who smoke receiving NRT, with 95% Confidence Intervals (CI); p-values	Prevalence of 18% of people who smoke using vapes had abstained from smoking	<0.001
				1.75	0.001 95% CI (1.24 – 2.46)
	Kale *et al.* (2025a) ^72^	Adult (18+) smokers, receiving community treatment for any mental health condition. Exclusions included those who already regularly using vapes.	Percentages (%) for abstinence. P-values not the primary focus for efficacy due to feasibility design. Very low n (this is 2/21 of e-cig group having confirmed abstinence after a month vs 0/22 for usual care)	Prevalence of 9.5%	One-sided 80% CI upper bound = 15%
	Jackson *et al.* (2022) ^73^	Smoking Toolkit Study	Adjusted Odds Ratios (OR) from logistic regression	1.12	NS (P = 0.489) 95% CI (0.82 – 1.53)
Regular Smoker/Regular Vaper to Smoking Quitter/Regular Vaper	Simonavicius *et al.* (2020) ^64^	Online survey of UK panel members (Ipsos MORI)	Used latent transition analysis to estimate probability of moving between classes for a 16 month periods	Transition Probability of 11.4%	Not given
	Jackson *et al.* (2025)	Smoking Toolkit Study	Adjusted Odds Ratios (ORadj) with 95% CIs; Bayes Factors (BFs) of quit for those who used vapes versus those who did not	1.95	P < 0.05 95% CI (1.74 – 2.17)
	Hardie & Green (2023) ^68^	UK Household Longitudinal Study (UKHLS)	Odds Ratios (OR) derived from weighted Marginal Structural Models	1.13	NS 95% CI (0.82 – 1.55)
	Kale *et al.* (2025b) ^71^	Adults (18+) either 'cigarette smokers only' or 'dual users' at baseline. Sample was young (mean age ~25)	Odds Ratios (OR) from logistic regression (adjusted for age and gender), for quit for at least 1 month at 3 months for those using vapes versus those that did not	5.16	P < 0.01 95% CI (1.09 – 24.41)
Ex-smoker/Regular Vaper to Regular Smoker/Regular Vaper	Simonavicius *et al.* (2020) ^64^	Online survey of UK panel members (Ipsos MORI)	Used latent transition analysis to estimate probability of moving between classes for a 16 month period	7.10%	Not given
	Hardie & Green (2023) ^68^	UK Household Longitudinal Study (UKHLS)	Odds Ratios (OR) derived from weighted Marginal Structural Models for relapse of people who self reported as ex-smokers =who used vapes versus those that did not	2.97	P = 0.05 95% CI (2.10 – 4.22)
Ex-smoker/Regular Vaper to Regular Smoker/Non-Vaper	Simonavicius *et al.* (2020) ^64^	Online survey of UK panel members (Ipsos MORI)	Used latent transition analysis to estimate probability of moving between classes for 1 16 month period	Transition Probability of 1.6% (Heavy Smokers)	Not given
				Transition Probability of 3.8% (Light Smokers)	Not given

There is consistent evidence from one UK study ^54^ and two England focussed studies ^45, 55^ that vapes are used by adults attempting to quit smoking and are associated with higher success rates compared to attempts without vapes or sometimes compared to NRT use, especially in supported settings or with daily use ^3, 64, 65^. One UK study showed that around 14% of young people who vape go on to smoking after one year; however, it is not known what the counterfactual would have been ^66^.

Evidence on relapse is less clear, with a UK study reporting that while daily vaping showed similar relapse rates to not vaping, infrequent vaping increased the chance of relapse, while there was also variation in relapse across the different kinds of vapes used ^67^. Another study found similar relapse rates for people who formerly smoked who vape versus those who do not ^64^, while others suggest potential risks, particularly for people who formerly smoked initiating vaping ^68^.

Of the studies identified, those by Moore, *et al.* ^69^, Simonavicius, *et al.* ^64^, Jackson, *et al.* ^3^, Jackson, *et al.* ^70^, Kale, *et al.* ^71^ and Hardie and Green ^68^ were considered to be most useful for informing a model of vape policy. In particular, the study by Simonavicius, *et al.* ^64^ provided detailed insights into movements between multiple behavioural states. This UK longitudinal study employed Latent Transition Analysis (LTA) on data from an online survey of UK adults (recruited via Ipsos MORI panel, quota sampled) who had smoked in the past year at baseline in 2016 (n=2857 at baseline, n=1471 followed up in 2017). Its primary aim was to identify distinct underlying groups (latent classes) based on patterns of smoking, vaping, nicotine replacement product (NRT) use, smoking urges, and quit attempts, and then to map the transitions between these groups over an approximate 16-month period. This study provides valuable UK-specific data using a sophisticated method (LTA) to show that transitions are complex and pathway-dependent. While alternative nicotine products (NRT and vapes) used by people who smoke are associated with higher chances of transitioning away from smoking compared to people who smoke not using aids, the specific endpoint differs (NRT users moving more towards complete abstinence, vapers moving more towards vaping ex-smoker status). People who smoked and used vapes had the highest probability of quitting smoking. The stability of vaping among people who formerly smoked and the similar relapse rates between people who formerly smoked and who did or did not vape are also key findings. However, it should be noted that this cohort study has now ceased and transitions between smoking and vaping behaviours will change over time.

Specific evidence related to priority subgroups of interest was limited among the UK evidence identified:

- **Young People (UK):** Findings suggest vapes are associated with cessation in young adult dual users (Kale D 2023, AOR 5.16) ^71^. Age-stratified analysis indicates vapes aid cessation significantly in the 18–64 age group ^55^ but non-significantly in the 65+ group. Trend analysis around Tobacco Products Directive (TPD) did not find significant changes in regular vaping initiation among never-smoking adolescents in England ^69^.
- **Mental Health (UK):** One UK feasibility RCT ^72^ involving adults receiving community mental health treatment found that providing a vape starter kit resulted in a 9.5% validated short-term (1-month) smoking abstinence rate, compared to 0% in the usual care group. While positive, the study was too small for conclusive efficacy findings.
- **Socioeconomic Status (SES):** No studies included presented quantitative findings/transitions stratified by SES indicators (e.g., income, education). This remains a significant evidence gap.

**Policy impacts on transition probabilities**

Two UK studies were identified that explored the impact of vape policies on smoking and vaping transitions ^69, 74^. These studies focused on the effect of Tobacco Products Directives for youth and adults as shown in Table 3 below.

**Table 3.** Studies showing policy effects in the UK context.

Specific Policy	Paper	Effect of Policy
TPD (Packet warnings, advert restrictions, nicotine strength limits) on youth e-cig use	Moore *et al.* (2020) ^69^	Potential slowing/plateauing of ever vaping uptake in young people (Wales); mixed effects on regular use. Limited youth awareness.
TPD (E-liquid volume, tank size, nicotine concentration limits) on adult vapers	Lee *et al.* (2020) ^74^	Increased use of TPD-compliant products; these specific TPD restrictions were not associated with an increase in smoking among adult vapers post-implementation.
TPD: Tobacco Products Directive		

Further research is needed to clarify the relative impacts of different vaping policy types in the UK context and to specifically investigate differential effects across key subgroups, particularly people with mental health issues. One point to note here is that some changes in behaviour may precede actual policy implementation (e.g., as recent research has shown for the ban on disposable vapes) ^75^, something which was also observed for tobacco policies (e.g., smokefree legislation) ^76, 77^. This highlights the need for careful statistical analyses and sensitivity analyses to consider a range of possible impacts.

**Quantitative evidence on the influences on smoking and vaping behaviours**

**Figure 11.**
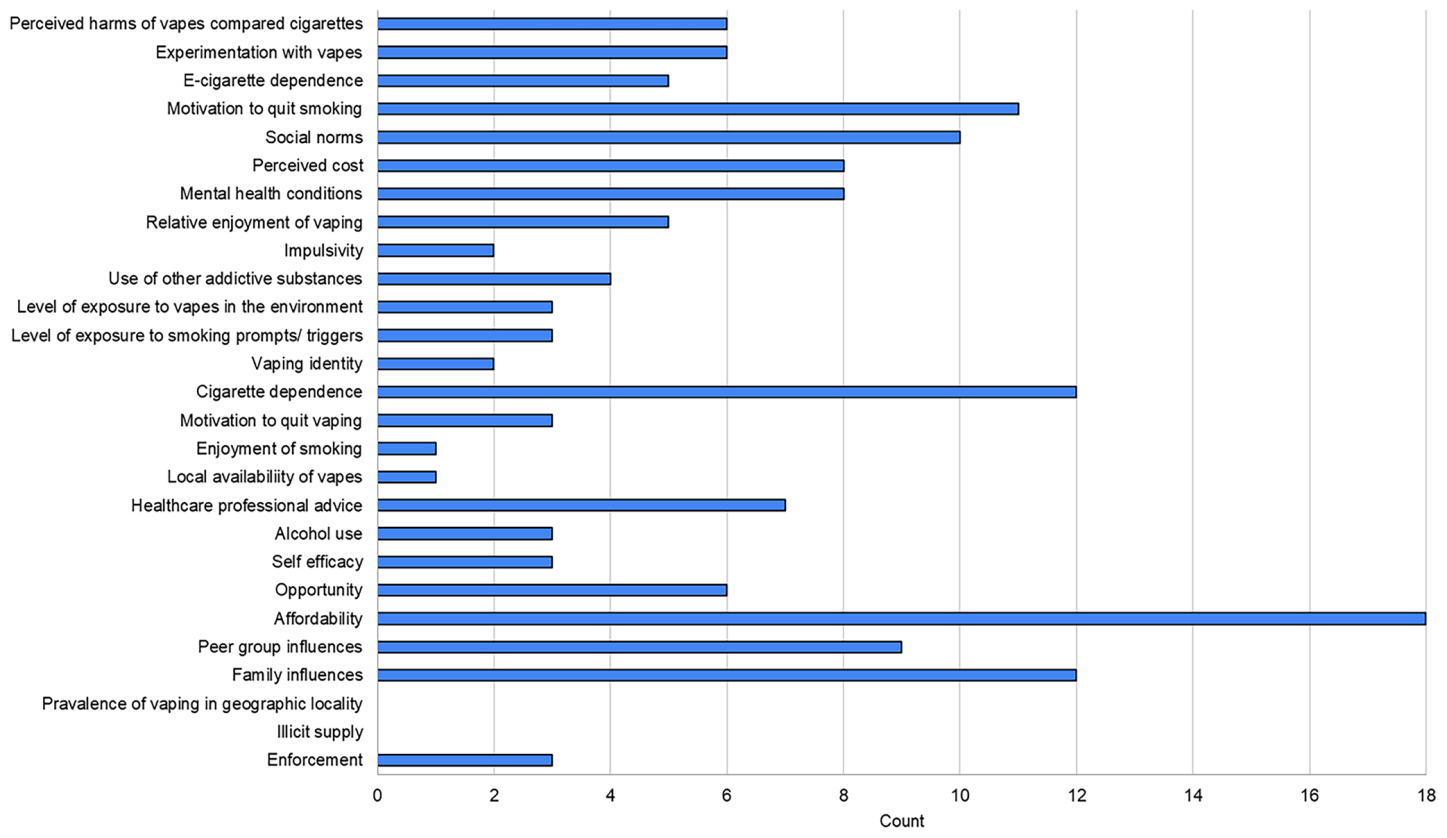
Number of studies reporting quantitative evidence on the influences of vaping and smoking included in the behavioural systems maps.

Twenty-five studies ^71– 73, 76, 78– 98^ were identified which presented some quantifiable evidence around influences on smoking and vaping behaviours. Figure 11 presents the number of studies reporting on each of the influences included within the behavioural systems maps developed within the workshop (see Figure 3– Figure 5).

Eighteen of the studies reported on how affordability affects vaping and smoking behaviours, suggesting that this is an important influence on behaviour. The evidence suggests that other key influences on smoking and vaping behaviours include family influences, peer influences and social norms; cigarette dependence; motivation to quit smoking; mental health conditions; and perceived harms of vaping compared to smoking. After our searches were undertaken, a further study was published assessing the impact of harm perceptions on smoking and vaping behaviours using longitudinal UK data, which showed that the perception that vaping is less harmful than smoking was associated with stopping smoking and now vaping ^99^. Within the second workshop the importance of perceived harms on behaviours was re-emphasised, with stakeholders suggesting that people’s beliefs about the extent of vaping versus smoking harms will overwhelmingly impact their behaviour and the effect of policies, and these are heavily influenced by the media. Stakeholders also highlighted that these beliefs have changed and will continue to change over time and are different between the four constituent countries of the UK.

While the current data indicates limited coverage for "experimentation with vapes," this number could potentially be expanded by considering measurements of "ever use" as indicative of initial experimentation. Notably, two factors, "illicit supply" and "prevalence of vaping in geographic locations," were not directly addressed by any included studies. The absence of data on illicit supply is understandable due to the inherent challenges in its measurement. Regarding the geographic prevalence of vaping, although not directly measured, this aspect is indirectly linked to deprivation, social norms, peer group influence, and family influence – all of which are closely related.

***7.2.4 Review question 3: Industry responses to vape policy***

When policy is evaluated, there is a tendency to predict the effect without accounting for any changes made by industry in response as an attempt to maintain or increase their sales ^100^. This is challenging because it is difficult to predict industry developments and behaviours in response to new polices. However, if we can understand how industry has responded historically, it may give some insight about what they may do in the future. At present, the range of responses historically made by the vaping industry to policy changes has not been systematically documented and synthesised. We review the international literature due to lack of UK evidence across a range of policies. From our review, thirty-four studies examined industry responses (any response made by or initiated by the vaping industry) to vape policy, with the aim of undermining the policy and maintaining or increasing sales (see Online Table 1 available here: https://osf.io/8zaxc/). Policies examined included: vape bans ^101– 106^; ban on disposable vapes ^107, 108^; flavour bans ^104, 109– 117^; product definition ^104, 117– 122^; registration & authorisation ^107, 109, 123– 126^; restricting marketing ^111, 117, 127^; restricting the product ^104, 117, 128^; restricting retail (e.g., restriction to pharmacy, banning sale to minors) ^104, 119, 129– 131^; restricting the use location ^119, 132^; and policy more broadly ^101, 133, 134^.

Industry responses include lobbying, protesting and arguing against the policy (particularly from a public health angle but also using economic arguments), advertising and social media campaigns, reframing the product, introducing different products (e.g., bringing out disposable vapes in response to a flavour ban), adjusting the product specifications (e.g., size, price, nicotine content), illegally selling and promoting banned products, and producing hybrid products (e.g., disposable vapes with a removable battery). The industry entities responding included the giant tobacco corporations (BAT, PMI etc), manufacturers of vapes (but not tobacco products), industry associations, and specialist (vape shops) and non-specialist vape retailers. There were also industry-funded front groups, think tanks and user groups. The studies, with few exceptions, were very poor at quantifying the effectiveness of industry activity in undermining the effectiveness of policy. Even studies that provided quantitative evidence did not report on changes in use prevalence. In addition, the extent of industry's effectiveness may vary between jurisdictions according to, for example, the strength of local tobacco control advocacy in counter-campaigning. A summary of evidence by policy type is presented below:

- **Bans of all vapes:** None of the studies included said the bans had been repealed; in some countries (e.g., Mexico) vapes seemed widely available despite a ban; in other countries, industry’s only success was bringing attention to vapes (e.g., Singapore).
- **Bans of disposable vapes:** After ban implementation, disposable vapes were being seized at the border (in Australia) and ‘hybrid’ products which may technically fit the law, but may continue to be environmentally harmful, were on the market (in New Zealand). However, no included studies compared the market pre- and post- ban.
- **Ban of flavours:** Industry appeared to have neutralised the USA pod flavour ban particularly by introducing a new flavoured product (disposables) that was not covered by the legislation. The Chinese domestic flavour ban motivated companies to export flavoured products so the ban in China had impacts on markets elsewhere. Studies did not look at prevalence change pre- and post- flavour bans.
- **Product registration/authorisation:** Companies have continued to sell products despite not having authorisation. Products appear to have proliferated rather than been constricted by entry restrictions. Proliferation leads to more work for authorities reducing oversight. However, there were no studies comparing countries with different levels of restriction.
- **Restrictions in marketing:** Studies reported various incidents where companies had ignored restrictions and were asked to withdraw marketing but studies rarely reported whether they had complied. A few instances were reported where courts/legislation had sided with the company on marketing.
- **Restrictions in product characteristics:** European restrictions intended to reduce nicotine delivery (via capacity/nicotine concentration) have been overcome by companies. Studies did not report the average nicotine delivery or the extent to which this had led to dependence/increased prevalence.
- **Restrictions in retail:** Policies included bans on selling to minors, whilst 24% of vape shops in Southern California were reported as not displaying age of sale warnings. The overall effectiveness of restricting vape sale to pharmacies/prescription was not reported.
- **Restrictions on indoor use:** Industry campaigns hard against restrictions on indoor use in the US and Europe. Industry has had success in the US, but effectiveness was not reported in Europe.
- **General vape policy:** Several instances were described where industry has been able to control the policy narrative, insert industry-conducive language into draft legislation and successfully get politicians to table weak bills in parliaments. The success of these activities was not reported.

Papers retrieved by the research criteria gave a comprehensive account of the many ways in which industry attempts to undermine policy. Instances of effective policy undermined by industry were described; however, within the included papers, it was not possible to tell the overall extent that the policy had become ineffective. For example, a country with a disposable vape ban with some disposable vapes being illegally imported may still have fewer disposable vapes on sale than a country where there is no ban in force. One possibility to better understand effectiveness within future research would be to use quasi- and natural experiments to capture both the policy effect and the industry impacts on vaping and smoking behaviours.

There are key evidence gaps regarding our priority policies: price, prescriptive/product and place. Previous work has modelled the extent to which industry undermined tobacco taxation ^25, 135^. Methods developed for this could be replicated for vape taxes where they are applied and then used to model the effects of future vape taxes. A number of papers have considered prescriptive/product policies such as flavours and product registration. Such studies have provided detail of industry tactics such as introducing alternative products, which are quite easy to track in the UK as products have to be registered with the MHRA. Future studies would ideally link more directly to the impact of such tactics on sales and use prevalence. Many place restrictions have been introduced recently (e.g. restriction of sales to pharmacies or country wide disposables ban). Once these restrictions have had time to bed in, they require follow up to understand their impact on sales and prevalence.

### 7.3 Useful datasets for modelling, including strengths and limitations

Potential data sources to inform decision making can include population surveys (national cross-sectional and cohort data), market data (including sales, pricing, and advertising), and information from regulatory bodies tasked with the monitoring and enforcement of vaping regulations. Following the first stakeholder workshop, we developed a “dataset dictionary” spreadsheet to capture key information available within each of these datasets and how they can be accessed. The dataset dictionary is a living document that can be updated to include relevant information (see Online Table 2 available here: https://osf.io/8zaxc/) that currently includes thirty-four population surveys (nineteen cross-sectional and fifteen cohort study designs, including one national treatment service dataset), nine market datasets and five government data sources. In terms of regularity of data collection, the identified cross-sectional data sources (monthly n=1; quarterly n=1; annually n=9; greater than annually n=7) had more surveys with higher frequency data collection than the identified cohort studies (annually n=3; biennially n=1; greater than annually n=11) ( Table 4). In terms of key subgroups, 76% of surveys contained data on accepted measures of socioeconomic position, 50% contained data on self-reported mental health status, 32% contained data on a clinical mental health diagnosis. Regarding younger age groups, 30% of surveys focussed on 8–18 year olds, 33% on those aged 16 or older, 18% on those aged 18 or older.

**Table 4.** Summary of selected population survey data characteristics.

Survey	Design	Location	Representative	Collection frequency	Open access	Age	SEP measure	Mental health self-report
Adolescent Health Study (upcoming data set)	Cohort	UK	UK and nation	Other	No information	8-18	No information	No information
Avon Longitudinal Study of Parents and Children	Cohort	UK	No	Other	No	1+	IMD	Validated
Born in Bradford	Cohort	England	No	Other	No	16+	Latent class variable	Validated
English Longitudinal Study of Ageing (ELSA)	Cohort	England	Nation	Biennially	Yes	16+	Income	Validated
Evergreen life	Cohort	UK	No	Other	No	18+	No information	No information
Health Wise Wales	Cohort	Wales	No	Other	No	16+	Social grade	Non-Validated
International Tobacco Control Survey (youth and adult surveys)	Cohort	England	Nation	Annually	Yes	18+	Income	No
Millennium cohort study	Cohort	UK	UK and nation	Triennially	Yes	1+	Income	Validated
Our future health	Cohort	UK	No	Other	No	18+	Income	No
UK biobank	Cohort	UK	No	Other	No	40+	IMD	Validated
Understanding society	Cohort	UK	UK and nation	Annually	Yes	1+	IMD	Validated
Zoe Health Study	Cohort	UK	No	Other	No	16+	No information	Validated
Clinical Practice Research Datalink	Cohort	UK	No	Other	No	1+	IMD	Validated
Next steps	Cohort	England	Nation	Other	Yes	13+	IMD	Validated
Growing up in Scotland	Cohort	Scotland	Nation	Annually	Yes	1+	IMD	Validated
Action on Smoking and Health Smokefree GB survey (Adult)	Cross-sectional	Great Britain	UK	Annually	No	18+	Social grade	No
Action on Smoking and Health Smokefree GB survey (Youth)	Cross-sectional	Great Britain	UK	Annually	No	11–18	No information	No
Annual Population Survey	Cross-sectional	UK	UK and nation	Annually	Yes	16+	Social grade	Non-Validated
ASH Wales yougov survey	Cross-sectional	Wales	Nation	Annually	No information	18+	Social grade	No
ASH Youth Vaping Survey Wales	Cross-sectional	Wales	Nation	Other	No information	11–18	No information	No
Eurobarometer	Cross-sectional	UK	UK	Triennially	Yes	15+	difficulty paying bills	No
GP Patient Survey	Cross-sectional	England	Nation	Annually	No	18+	IMD	No
Health Behaviour in School-aged Children (HBSC)	Cross-sectional	Great Britain	Nation	Quaternally	Yes	11–15	Family affluence	Validated
Health Survey for England	Cross-sectional	England	Nation	Annually	Yes	16+	IMD	Validated
National Survey for Wales	Cross-sectional	Wales	Nation	Annually	Yes	16+	IMD	Validated
ONS Covid Infection Survey	Cross-sectional	UK	No	Other	No	1+	No information	No
Opinions and Lifestyle Survey	Cross-sectional	Great Britain	UK and nation	Monthly	Yes	16+	Income	Non-Validated
School Health Research Network (SHRN)	Cross-sectional	Wales	Nation	Biennially	No information	11–16	Family affluence	Validated
Scottish Health Survey	Cross-sectional	Scotland	Nation	Annually	Yes	16+	IMD	Validated
Scottish School Adolescent Lifestyle and Substance Use Survey	Cross-sectional	Scotland	Nation	Biennially	Yes	13–15	IMD	Validated
Smoking Toolkit Study	Cross-sectional	Great Britain	UK and nation	Monthly	Yes	16+	Social grade	Validated
Smoking, Drinking and Drug Use among Young People in England	Cross-sectional	England	Nation	Biennially	Yes	11–16	No information	No
The Schools and Students Health Education Unit: “Young People into” series	Cross-sectional	Great Britain	No	Annually	No	11–18	No information	No
Statistics on NHS stop smoking services	Cross-sectional	England	No	Quarterly	No	16+	Social grade	No

The identified population survey datasets (68% representative at the UK country or nation level; 47% available open access) contained varying coverage of data related to key smoking and vaping outcomes ( Table 5). The majority of studies contained basic data on current smoking status (currently smoking, formerly smoking, never smoked), but fewer contained data on vaping status or more detailed data to inform smoking and vaping transitions.

**Table 5.** Summary of coverage of key smoking and vaping outcome variables in identified population survey datasets.

Outcome domains	Variable name	Summary	Percentage of all surveys with data n (%)	Number of cross-sectional surveys with data (%)	Number of cohort surveys with data (%)
Smoking	Smoking status	Current; former; never	30 (88%)	16 (84%)	14 (93%)
	Recent former smoking	Stopped recently	9 (27%)	5 (26%)	4 (27%)
	Smoking quit attempt	Recent attempt to quit	9 (27)%	6 (32%)	3 (20%)
	Smoking quit maintenance	Remained quit following attempt	7 (21%)	5 (26%)	2 (13%)
	Motivation to stop smoking	Motivation to stop soon	10 (29%)	8 (42%)	2 (13%)
Vaping	Vaping status	Current; former; never	22 (65%)	13 (68%)	9 (60%)
	Vaping experimentation	Ever tried vaping	18 (53%)	16 (84%)	11 (73%)
	Recent former vaping	Stopped recently	2 (6%)	1 (5%)	1 (7%)
	Vaping quit attempt	Recent attempt to quit	1 (3%)	0 (0%)	1 (7%)
	Vaping quit maintenance	Remained quit following attempt	1 (3%)	0 (0%)	1 (7%)
	Motivation to stop vaping	Motivation to stop soon	1 (3%)	1 (5%)	0 (0%)

It should be noted that the Health Survey for England is due to cease in its current form which is an important loss; it provides high quality data for key variables across the population of England, including measuring cotinine levels which provide an objective measure of nicotine intake. In addition, the funding for the ITC study is under threat. Advocacy is needed for the support of these studies.

The identified market datasets are summarized in Table 6, and include global market research data, point of sale data, and UK government agency data.

**Table 6.** Summary of identified market research data and relevance for vape policy modelling.

Dataset	SPIRE Policy relevance	Price data	Sales data	Product characteristics	Market share	Advertising
Euromonitor	Price Place	Yes	Yes	Brand Type Flavour Nicotine level	Yes	No
Nielsen *	Price Place	Yes	Yes	No	Can be calculated	Expenditure
Ecigintelligence	Price Place Prescriptive	Yes	Unclear	No information	No	No information
Kantar	Prescriptive	No	Yes	Brand Type Flavour Nicotine level	No	Expenditure Exposure
Retail Data Partnership (shopmate)	Price Place	Yes	Yes	Brand Type Flavour Nicotine level	No	No
Medicines and Healthcare products Regulatory Agency (MHRA) registrations	Prescriptive	No	No	Brand Type Flavour Nicotine level	No	No
HMRC Tobacco Bulletin **	Price	Yes	No	No	No	No

*Only collects data from general retailers – does not include vape shops or online **Only collects data on tobacco products (not vaping products)

Other datasets deemed relevant for vape policy modelling include the UK government data from the Medicines and Healthcare products Regulatory Agency (MHRA), Yellow card scheme analysis prints (detailing adverse reaction or safety concerns about vapes and/or e-liquid), underage test purchases, tobacco mass media expenditure, tobacco and vaping product duty rates, tobacco and vaping product affordability indices and Trading Standards reports on illicit product seizures.

At the local authority level there are also school-based surveys (Health Related Behaviour Questionnaires) which could be useful; however, they currently lack consistency.

**New trial registrations and funded projects**

The ISRCTN registry (includes all interventional and non-interventional clinical studies that prospectively involve UK participants and evaluate biomedical or health-related outcomes), and leading funder databases known to fund research in this area (NIHR, CRUK, MRC, BHF) were searched on 29/4/2025 for ongoing RCT registrations and research projects related to vaping and smoking. This ISRCTN search returned two ongoing trials, one assessing the effect of vapes for smoking cessation and reduction in people with a mental illness ( https://doi.org/10.1186/ISRCTN14068059) and the other assessing the effectiveness of electronic cigarettes compared with combination nicotine replacement therapy for smoking cessation in patients with chronic obstructive pulmonary disease and effect on lung health ( https://doi.org/10.1186/ISRCTN82413824). In addition, a new £60 million Adolescent Health Study with a focus on vaping was recently funded, which will collect data on 100,000 youth over a 10 year period ( https://www.gov.uk/government/news/10-year-study-to-shed-light-on-youth-vaping).

### 7.4 Key non-UK evidence that could supplement the UK evidence

***7.4.1 Key non-UK evidence on vape harms***

 As mentioned in Section 7.1.1, the majority of studies assessing the absolute and relative health risks / harms of vaping have been conducted in non-UK settings. Other systematic reviews by international colleagues published more recently build on and extend the findings reported by McNeill *et al.* and the Royal College of Physicians report on vapes. A systematic review and meta-analysis on the cardiovascular effects of vapes by Kundu *et al.* ^136^ builds upon and extends the findings of the DHSC commissioned McNeill *et al.* This expanded evidence base allowed for more robust subgroup analysis. Overall, the review included 63 studies, and the authors concluded that acute vaping increases cardiovascular stress (due to nicotine exposure), short-to-medium term switching from cigarette smoking to vaping may improve endothelial function and blood pressure but there was no increased incidence of cardiovascular disease. Six of the studies included a never smoking group of vape users (where any changes would be related to vapes not former smoking) and found similar results. Acute vaping exposure studies (three studies) found heart rate and blood pressure increased only during and proximal to exposure, whereas there was no increased risk of cardiovascular markers with longer vaping exposures (up to six years) in a further three studies. With more good quality studies including people who have never smoked, it is likely we will be able to model the short, medium and long term absolute and relative effects of the cardiovascular risks of vaping.

In another systematic review and meta-analysis by the same research group on cancer risk and vapes ^137^, 12 studies in humans were included, eight of which included people who have never smoked. Across all the studies involving people who have never smoked there was no clinical evidence of cancer in people who have never smoked who vape. There were some biomarker and epigenic changes in oxidative stress, inflammation and DNA damage. Whilst these do not equate to disease the authors suggested they may indicate biological plausibility for future risk. Exposure periods were short and as cancer has a long latency period, longer term follow up data are needed to better understand cancer risk among vapers who have never smoked.

Another systematic review of 10 prospective human studies examining respiratory health outcomes in people who vaped but who had never smoked found vape use was not associated with significant respiratory disease or clinically meaningful symptoms over 1–5 years ^138^. There was some signal for mild symptoms (e.g., cough or wheeze), but no clear evidence of harm from objective lung tests or disease incidence. No meta-analyses were included due to heterogeneity in study design, exposure definitions and outcome measures, suggesting it may be difficult to model respiratory studies based on the current evidence.

Finally, several large reviews reported additional negative effects of vaping. One umbrella review found some acute cardiopulmonary risks yet long-term use may have some respiratory benefits for smokers who switch to vaping, with little evidence available on carcinogenic effects ^139^. Another meta-analysis reports that there is increased risk compared with non-use and limited evidence of improvements among vapers compared with smokers for cardiovascular disease, stroke or metabolic dysfunction (but some improvements for asthma and COPD) ^140^. Similarly, another umbrella review concludes that e-cigarettes can be harmful to health, which also highlight concerns (discussed in more detail in 7.1.2) about the impact of exposure of children and adolescents to nicotine in vaping solutions ^51^, leading to long-term negative impacts on brain development as well as addiction ^141^. However, it should be noted that the latter two reviews have been critiqued for using inappropriate methodology to arrive at these conclusions ^142, 143^.

***7.4.2 Key non-UK evidence on the transition from vaping to smoking***

Given the increasing use of vapes by people who do not smoke ^7^, there are concerns that vapes may act as a transition to later regular smoking, especially among youth ^144^. The so-called “gateway hypothesis” has been criticised as it originated from work about transition of use of one drug to another, whereas in the context of vaping and smoking the same drug is used (nicotine). Further, discussions often focus on a gateway into smoking when the opposite is also possible (vaping as a gateway out of smoking), something which cannot be easily evaluated with individual-level observational studies as a gateway out would require counterfactual scenarios to be evaluated ^145^. Our reviews identified only one UK study on this transition, yet our review of simulation models suggested that model results can be sensitive to the assumptions made around these parameters. Several international studies and systematic reviews have been published about the transition to regular smoking from vaping ^146– 149^. While longitudinal studies of individuals show that young vapers are more likely than non-vapers to go on to start smoking ^146^, these studies have been criticised for not being able to account fully for relevant confounders and the fact that vaping and smoking may be driven by common factors rather than one causing the other ^147, 148^. Indeed, repeat cross-sectional population-level studies (mainly from the US), which are not subject to the same problems, do not support the hypothesis that the increase in vaping among people who do not smoke has resulted in an increase in smoking rates ^146^. In fact, the results from ecological studies included in one review are more consistent with an interpretation that increasing vaping rates are associated with decreasing smoking rates ^146^.

***7.4.3 Key non-UK evidence on own- and cross-price elasticities of demand for vapes***

The effects of price interventions on the demand for vapes will depend on the characteristics of the consumer (e.g., smoking status, socioeconomic status, age) and on the vape product (e.g., type of vape, packaging, flavour, nicotine strength). However, there is a lack of evidence for the UK on how changes in vape price affect demand for vapes. Vape price increases may also have unintended consequences such as increasing cigarette smoking and growth of the illicit market.

Several US studies indicate that higher vape prices are generally associated with lower vaping. One study using retail data found that a 1% price rise would result in a 2.2–2.5% decrease in vape consumption ^28, 150^; another found that consumption would decrease by 1.2% for disposables and by 1.9% for reusables ^151^. However, this may not hold true across all populations (including queer youth) ^152^. Investigations in the US, in particular, have had the advantage of being able to utilise variation in vape and tobacco tax policy among states to investigate the effects of pricing policy changes on consumer behaviour. A recent study that took this approach found evidence that a 10% increase in vape taxes could reduce vape sales by 0.5% ^153^. Few studies have assessed the impact of price on youth vaping, but they consistently show that price increases reduce vaping, including vaping frequency and amount vaped ^154– 156^. As an unintended consequence, however, studies have also found that vape price increases increase cigarette use, including in adults and youth, pre-pregnancy and prenatal women, and queer youth ^28, 153, 155, 157– 160^. In the context of high levels of misperceptions of the risks of vapes versus cigarettes in the UK ^55^, there is a danger that increases in vaping duty may result in switching to cigarettes perceived as being equally or less harmful.

These studies used historical data, which have the advantage of showing what the real-life responses of past policy changes have been but might be considered insufficiently representative of the potential impact of future price interventions in rapidly developing markets such as vapes. Behavioural economic experiments which simulate purchasing behaviour provide a potential way of addressing these limitations – they can be setup to reflect the current market and current consumer preferences ^161^. They have been widely used to estimate the effects of price changes for tobacco, as well as the effects of prescriptive policies such as bans on menthol cigarettes ^162^, and several studies have now used this approach for vapes. This includes cross-commodity studies that investigate the effect of price and prescriptive policy changes on substitution with other products (e.g., switching from vapes to cigarettes) ^163^.

***7.4.4 Key non-UK evidence on company sales***

Transnational tobacco companies provide details on sales and profits in the Annual Reports (e.g., 164). They report overall statistics and also statistics by world region. UK data is merged into one of these world regions. Occasionally UK is mentioned in the text. The reports provide overviews of company priorities and can be useful to understand changes in products available in the UK.

## 8. Current evidence, data gaps and recommendations for vape policy modelling

### 8.1 Table of best available current evidence and gaps for each model requirement

Table 7 below summarises the main findings of the evidence gathering exercise, outlining the essential model requirements, what best evidence is currently available to address the requirements and what key gaps in current evidence remain.

**Table 7.** Available evidence and gaps in evidence.

Essential requirements	Best available current evidence	Key gaps in the current evidence
The transitions between vaping and smoking and the interactions between them over the lifetime of a group of heterogeneous individuals, including changes in prevalence of smoking and vaping over time (important individual characteristics include age, socioeconomic status and people with mental health conditions)	There is substantial UK evidence around many of the transitions, in particular around smoking cessation using vapes and quitting vapes as a person who formerly smoked. Simonavicius *et al.* (2020) provides detailed insights into movements between multiple behavioural states ^64^. Other key studies include Moore *et al.* (2020), Jackson *et al.* (2024), Jackson *et al.* (2025), Kale *et al.* (2023) and Hardie and Green (2023).	Consistent UK transitions across all behavioural states estimated simultaneously. Evidence across key subgroups including more disadvantaged socioeconomic groups and people with mental health conditions.
How policies will affect vaping and smoking behaviours	There is limited and mixed evidence about the impact of price, place and prescriptive policies in the UK on vaping and smoking behaviours.	UK evidence on the impact of vape policies on vaping and smoking behaviours in adults and young people.
The relative health harms associated with vaping compared with smoking and the absolute health harms of vaping compared to not smoking or vaping	Only one UK study reports on the harms of vaping for people who have never smoked. It shows no effect of vapes on DNA methylation profile associated with lung cancer ^45^. Two additional studies that include UK samples show higher levels for some biomarkers of exposure ^6, 45^. Several UK studies, and many US studies, assessing the relative effects of vaping versus smoking ^1, 167^. They suggest that vapers are exposed to significantly fewer harmful carcinogens and toxicants than people who smoke cigarettes. Based on the exposure profile resulting from vaping, estimates have been produced on the likely risks of developing smoking-related diseases such as lung cancer compared with cigarette smoking, putting this at between 1 – 7% ^168, 169^.	UK evidence on the health harms of vaping for people who have never smoked, including as it pertains to nicotine content in vapes (e.g., on compensatory behaviour as related to nicotine limits). Longer term evidence on the harms of vaping.
Desirable requirements	Best available current evidence	Key gaps in the current evidence
The influences on behaviour and the mechanisms of action of policies affecting vaping and smoking behaviours, including the interactions between socioeconomic factors, psychological factors, social networks, spatial factors (for “place” policies) and institutional, structural and cultural variables	26 UK studies were identified which quantified key influences on vaping and smoking behaviours. 9, 12 and 10 UK studies considered the impact on vaping of peer influences, family influences and social norms respectively. In addition, our dataset dictionary has datasets which include some of these influences, in particular the International Tobacco Control Survey (youth and adult), Action on Smoking and Health survey (youth and adult), the Avon Longitudinal Study of Parents and Children and the Smoking Toolkit Study (STS). The STS has collected one wave of data on smoking and vaping in close social networks. In addition, we identified substantial market research data reporting sales data, including Euromonitor, Nielsen, Kantar and Retail Data Partnership (shopmate).	Quantitative evidence linking illegal vape supply, experimentation with vapes and prevalence of vaping in geographic locations with vaping.
Price elasticities of demand for vape products and cross-price elasticities between vape and tobacco products (for “price” policies)	Several US studies indicate that higher vape prices are generally associated with lower vaping; however, there is no UK evidence.	UK evidence on own- and cross-price elasticities.
The potential impact on vaping and smoking behaviours of industry responses to government policy	There are many studies that give a comprehensive account of how companies attempt to undermine a variety of policies, However, there is very limited quantification of the effectiveness of these activities in undermining policy and policy goals.	Quantifying the impact of industry activity undermining policy on vaping, prevalence and sales. Studies of counterfactuals, for instance estimated impact of a policy if industry had not interfered compared with actual impact of the policy. Evidence on industry activity regarding vape taxation and plain packaging.
The additional harms of use of illegal vapes (non-compliant/containing illicit substances) compared with use of legal vapes and prevalence of use	There is limited evidence directly comparing the impact of legal vs illegal vapes. It is likely that illegal vapes carry a bigger risk than legal vapes, given the lack of oversight and regulation of what they contain, but there is currently no quantifiable evidence. Availability of illegal products is also mainly limited to small convenience samples. The ASH 2024 survey included questions about puff count and nicotine concentration and Trading Standards reports on illicit product seizures, suggesting recent increases.	Quantifiable evidence on the additional harms of use of illegal versus legal vapes and accurate data on use.
The interaction between vaping and use of other products for smoking cessation	A study by Jackson *et al.* has shown that the increase in vaping for smoking cessation in the UK has resulted in an increase in the use of any smoking cessation aid rather than a decrease in the use of other smoking cessation aids ^3^.	How the use of nicotine pouches might interact with vape use in the UK; evidence on the relative harms of pouches compared with vapes, and the relative effectiveness for smoking cessation (including co-use with other products such as nicotine replacement therapy).
Smoking and vaping dual use outcomes	The impact of dual use of vapes and cigarettes on exposure to harmful constituents depends on the patterns of dual use. Add-on use with vapes is unlikely to carry health benefits, while displacement use (resulting in lower cigarette consumption) may do ^47^. In general, the literature shows large heterogeneity, making general statements about dual use problematic ^48^.	Better classifications of dual use are needed to estimate impact on health and transitions between dual use and cessation.
Environmental outcomes associated with vapes	*Outside of this study scope*	Further research is required to identify available evidence
Retail outcomes (convenience store footfall) associated with vapes	*Outside of this study scope*	Further research is required to identify available evidence
The costs and QALYs associated with new policies	Most existing simulation models predict net benefits from vaping policies; however, some do not. There are only two models that have been applied to the UK context (Levy *et al.* ^13^ and Kalkhoran *et al.* ^12^), and neither of these models would allow the analysis of the impact of vape policy on important subgroups of the population.	Evidence in the form of a UK individual-level simulation model of vaping policies.

### 8.2 Recommended additional survey questions and primary research to facilitate vape policy modelling (short, medium and longer term)

There is currently good coverage of detailed smoking and vaping data in several national cross-sectional surveys in the UK (Section 7.3) with varying regularity of data collection (monthly to annually). There is a need for standardisation of measures for smoking and vaping across datasets, for example measures for ‘regular use’ and ‘ever use’. Additional survey questions that are not currently or routinely collected in national cohort surveys, and which would be important to inform vape policy modelling, are summarised in Table 8 below.

**Table 8.** Recommended additional survey questions.

Study design	Measure	Rationale for addition
Cohort	Frequency/type of vaping	Only two cohort surveys (ITC project * and Understanding Society) collect these data annually, and it is restricted to England only. Important to distinguish regular use from infrequent use to inform understanding of benefits for smoking cessation, and longer-term harms from exposure at the UK level. For youth in particular, it would be useful to gather information on sharing behaviour of vapes as this has been anecdotally observed.
Cohort and repeated cross-sectional	Vape device characteristics (flavours, nicotine concentration, device types, legal vs illegal) and vaping cessation	Only one cohort survey (ITC project *) collects these data annually, and it is restricted to England only. Provide information to inform impact of policy on device use, and in relation to smoking behaviour and smoking cessation at the UK level, including on type of nicotine used (freebase, salts) and use of products that are non-compliant or contain illicit substances.
Cohort	Age of smoking cessation in those who formerly smoked	Allows inference on potential impact of vaping policy on returning to smoking according to time since quit.
Cohort	Collection of biomarkers of exposure or potential harm	Few cohort studies collect matrices (urine, blood) that can be analysed to assess potential harm of product use (e.g., Our Future Health); addition of sample collection to other existing cohort studies will be essential to allow evaluation of likely harms of new products coming to market (including illegal products).
Cohort	Standardised mental health measure	Data on the relationship between vaping and mental health are scarce.

*Due to recent changes in the US funding landscape, the ITC project is likely to be defunded. Given the importance of the dataset to UK policy research, UK funding should therefore be provided to bridge this gap.

**Recommendations for primary research are listed below:**

*Short term*

1. Longitudinal studies that measure detailed smoking and vaping behaviours and their sociodemographic and mental health correlates, with frequent data collection that permit capture of transitions between behaviours in relation to policy, along with consistent collection of samples for biomarkers of harm.
2. Studies (experimental lab studies or nested within large population surveys) using the Experimental Tobacco Marketplace (ETM) methodology ^165, 166^ to forecast the impact of policy on tobacco product purchasing.
3. Run focus groups with users to test survey questions about assessing the size and nature of the unregulated market.

*Medium term*

4. A set of experiments (natural, quasi, behavioural) to assess the impact of vape policies in the UK setting.
5. Randomised controlled trials on the relative effectiveness of nicotine pouches compared with vapes for smoking cessation, and the relative harms.

*Longer term*

6. Longitudinal studies that measure detailed smoking and vaping behaviours and their correlates, and physical health outcomes or link health record and mortality datasets, e.g., to allow comparison between users and non-users of products and of users of regulated vapes with users of unregulated vapes (non-compliant nicotine vapes/vapes containing illicit substances).

**Recommendations for other data collection are listed below:**

HMRC provides a Bulletin ( Tobacco Bulletin - GOV.UK) detailing the volumes and value of tobacco products cleared for sale in the UK for tax purposes and additionally information on the tax gap; in short, the gap between taxed products and total market size can be used to estimate the size of the illicit tobacco market. With the onset of the vaping product duty in October 2026, the government should be encouraged to produce similar information for vapes.

Standardisation of local authority level school-based questions in Health-Related Behaviour Questionnaires at a national level via co-creation with schools and collation of these data would be beneficial for modelling at the local and national level. In addition, data collection on illicit products at a more local level could help identify where limited resources for enforcement and measurement should be allocated. Local level data is also helpful for considering the impact of nicotine product purchasing on poverty in lower-income groups, especially as retailer licensing occurs at the local level. It would be useful to map how local level data links together and establish common Data Sharing Agreements. This is particularly important given differences in health data collection across the four nations of the UK. In addition, more nation-disaggregated data would be preferable, including more data collection for Northern Ireland.

### 8.3 Recommendations for modelling vape policies

***8.3.1 Calibrate life course dynamics of vaping and smoking behaviour***

 The review of existing modelling studies (Section 5.2) showed how previous studies have used incomplete transition diagrams, with the two UK modelling studies including only tobacco smoking transitions which could be altered by vaping. Review question 2a showed that there is currently good UK evidence about specific transition probabilities, but no coherent system of transition probabilities across joint vaping and tobacco behaviours. We recommend using these previously estimated transition probabilities as initial parameters for a calibration to estimate a coherent system of transition probabilities across joint vaping and tobacco behaviours for a UK baseline population. In the small number of cases where limited UK evidence exists on transitions, this could be supplemented with international evidence for the priors. Calibration targets can be formed by the joint prevalence of smoking and vaping e.g., the percentage of people who have never smoked and currently vape. These can be taken from existing surveys including the Smoking Toolkit Study, ASH data, the Health Survey for England, Scottish Health Survey, National Survey for Wales, the International Tobacco Control Survey, Understanding Society, the Avon Longitudinal Study of Parents and Children and the Millennium Cohort Study. We recommend that this calibration should estimate age-specific transition probabilities for each state and use time trends in these transitions given the change in prevalence of smoking and vaping over time.

This calibration process for behavioural transitions could also be applied to key priority subgroups i.e. according to socioeconomic status and for people with mental health conditions.

***8.3.2 Precisely define specific policy options and describe their mechanisms of action, generating new evidence from models***

 The workshop identified the three priority policy themes as price, prescriptive and place (see Section 3.1). Note that in future work we would use a different term for prescriptive policies as some of the stakeholders found this term ambiguous. Within these broadly defined themes lie a variety of policy options that a model could be used to appraise, in terms of their different effects on vaping and smoking behaviour. Before developing the detailed mechanisms of specific policy-to-behaviour modules, it is important to define the policy options currently “on the table” and for which the policy deliberations could be supported by modelling. For example, see Hatchard *et al.* ^170^, which developed a specific understanding of pricing policy options across tobacco and alcohol, deepening the more general description across multiple policy themes in Gillespie *et al.* ^20^ A behaviour change intervention ontology could be used to precisely specify the interventions which will allow for better integration of data and evidence ^27^. Taking a complex systems approach could support understanding of how specific policy options within a theme, e.g., increasing the rate of tax on e-liquids, might interact with specific policy options from another theme, e.g., a ban on certain e-liquid flavours. The interactions with tobacco policy (e.g., age of sale) should also be considered.

There are generally two approaches to specifying how a particular policy option will affect behaviour: (1) Using effect sizes estimated from evaluations of previous relevant policy changes; (2) Building a policy module that specifies the step-by-step mechanisms for how a policy change is transformed to a change in behaviour. Given that the review (see Section 7.2.3) found limited UK evidence that could be used for (1), the recommended approach is (2), developing mechanistic model structures, linking each step in the mechanism to best-evidence and testing key assumptions (see 8.3.4).

Modelling of the effects of different types of policy on vaping and smoking behaviour is likely to share common components, e.g., the life course dynamics of vaping and smoking behaviour, which can be informed by UK evidence (see 8.3.1). However, the general lack of UK-specific evidence for how consumers might respond to new vape regulations is concerning. For example, there is limited UK-specific evidence on the price elasticities of demand for vapes (price), the effect of flavour bans (prescriptive), or new retail licensing rules (place). For this reason, it is recommended that modelling projects also include the generation of new evidence for these key steps in estimating policy effects, with model structures developed to accept this new evidence and to explore the influence of any remaining uncertainty.

***8.3.3 Consider the impact of industry influence***

 Review question 3 demonstrates the high prevalence of industry reactions to government policy, which in some cases have partially or completely mitigated policies. In particular, flavour bans, disposable vape bans and restrictions regarding nicotine delivery have been circumvented by industry by introducing new products that are not strictly covered by existing law. Flavour and probably nicotine delivery focused policies appear to have been effectively nullified (but more evidence on vape prevalence would be needed to confirm this). It is challenging to incorporate industry influence into a model because of the uncertainty associated with industry reaction to government policy and the limited evidence available about the effectiveness of any policy mitigation by industry, but this has not been the case for industry manipulation of tax passthrough, e.g. for tobacco and alcohol ^171^. We recommend that all vape policy models use the results of review 3 to consider potential industry action to mitigate specific vape policies, and for this to be as a minimum noted within modelling reports, and ideally for exploratory analyses to be undertaken to assess possible effects.

***8.3.4 Quantify the mechanisms of action of policies and other influences, including industry and media, on smoking and vaping behaviours***

 It is difficult to predict the longer-term effects of policies if there is limited understanding of their mechanisms of action. Modellers should identify which theories (if any) were used to develop the interventions. If resources allow, we recommend undertaking causal mapping for each policy to be assessed to describe the mechanisms of the policies that impact smoking and vaping behaviours. This should be integrated with other impacts on these mechanisms and behaviours including media and industry influences, given the importance of perception of relative harms on smoking and vaping behaviours and industry circumvention. For example, see the mechanisms developed to link pricing policy options to tobacco and alcohol consumption by Morris *et al.* ^171^ Those mechanisms use market research and consumer spending diary data to establish the baseline distributions of the prices of products bought by consumers ^172^, and then model how the price distributions are expected to change following a policy change, considering the potential for industry to modify the intended policy effects, e.g. by reducing their profits from the sale of some products ^173, 174^. The resulting change to the average prices faced by consumers is then translated to a change in the consumption of each product using estimated price elasticities of demand ^175^. It is also possible to include individual psychological variables; for example, people’s beliefs about the extent of vaping versus smoking harms will impact their behaviours and the effect of policies. This can be quantified using theory-based statistical analyses such as structural equation modelling and survey data such as the Smoking Toolkit Study and the International Tobacco Control Survey which collect psychological variables and other influences on behaviour (e.g., see for example Tian *et al.* ^30^). These analyses could be combined with agent-based modelling to help describe and predict the impacts of policies upon smoking and vaping behaviours.

With regards to the impact of policy, some UK evidence would suggest that restricting nicotine content may result in compensatory (more intense) puffing to achieve desired levels of nicotine intake, with the unintended consequence of increasing exposure to BoE/BoPH ^176^. However, given the currently limited UK evidence on the impact of e-cigarette policies, international evidence could be drawn upon initially, including from Europe and Canada. It should be noted that, given the differences between the UK and other countries in terms of tobacco and vaping policies and smoking and vaping prevalence, there will be substantial uncertainty around the generalisability to the UK setting which should be quantified within a model. We also recommend further experimental primary research in the UK about policy effects.

Over the longer term, we recommend undertaking social network analysis and incorporating this within an agent-based model, given that smoking and vaping behaviours have been shown to be influenced by others within an individual’s network ^83^. For place-based interventions we recommend considering incorporating spatial analysis within an agent-based model.

***8.3.5. Prioritise including the uncertainty in vaping harms in people who have never smoked over modelling the harms of vaping for dual users or former smokers that vape***

a) Harms of vaping in people who have never smoked

There is very limited UK evidence around the harms of vaping in people who have never smoked. However, international evidence suggests that people who vape are exposed to significantly fewer harmful chemicals, carcinogens and toxicants than people who smoke cigarettes ^1, 167^. Based on the exposure profile resulting from vaping, estimates have been produced on the likely risks of developing smoking-related diseases such as lung cancer compared with cigarette smoking, putting this at between 1 - 7% ^168, 169^. This is consistent with long-term evidence on snus use from Scandinavia, which generally shows that detrimental effects are limited ^177, 178^.

Given the known bidirectional effects of cigarette use (and addiction more generally) on mental health problems, which may, in part, be mediated by nicotine ^179^, taken together with increasing levels of addiction to vapes reported by young people ^180^, there are concerns about the mental health effects of vaping in people who have never smoked, and it will be important for models to consider these outcomes, and incorporate the current uncertainty around them. We recommend that further research is needed to estimate vaping harms in people who have never smoked in the UK setting, including the effects of nicotine addiction on young people’s health and wellbeing, given limited evidence in humans.

b) Harms of vaping in people who smoke

This is a heterogeneous group; for instance, daily use of vapes with non-daily cigarette smoking does reduce exposure, but daily use of cigarettes with non-daily vaping does not, even if the number of cigarettes smoked per day is reduced. It would be challenging to incorporate this level of complexity into a model and the harms of different levels of dual use are currently unclear. With the goal of parsimony, we recommend provisionally assuming the harms of dual use are equivalent to the harms of smoking, as current evidence suggests that the smoking harms dominate.

c) Harms of vaping in people who formerly smoked

There is currently limited evidence on the harms of vaping in people who formerly smoked. As above, given that smoking harms dominate over vaping harms, we recommend provisionally assuming the harms of vaping are negligible for people who formerly smoked relative to the harms of being a former smoker.

***8.3.6. Collect more evidence on the use of illegal vapes for incorporation into future modelling***

 There is currently a lack of sufficient data on the market for illegal vapes (i.e., nicotine vapes which do not comply with regulation, vapes containing illicit/banned substances such as Class A drugs) and on illegal practices (sale of legal or illegal vapes to minors), including the prevalence and harms of this use compared with legal vape use, so it would be challenging to incorporate this within a simulation model. Developing this evidence should be a priority; stakeholders agreed that the use of illegal vapes is likely to be an unintended consequence of several vaping policies. There are two key questions: 1) Whether and to what extent illegal products are more harmful than legal products; 2) The extent to which vaping policies encouraging switching to illegal products. The use of experimental tobacco marketplace approaches can yield further insights into the latter, as they can be designed to include both illicit and licit markets. Additional questions in existing surveys on the use of non-compliant vapes and vapes containing illicit substances may also provide a better picture of prevalence of use of illegal vapes, and potentially about health consequences if linked to health care records.

***8.3.7. Model the interaction between the use of nicotine pouches and vapes if the trend in use of nicotine pouches in the UK continues to increase***

 Evidence suggests that in the UK the use of vapes for smoking cessation has had minimal impact on the use of other nicotine products ^181^. Nicotine pouches are a relatively recent addition to the global tobacco and nicotine market and their use in the UK is markedly increasing ^182^. It will therefore be preferable, if feasible, to include the interaction between the use of nicotine pouches and vapes if the trend in use of nicotine pouches continues to increase.

***8.3.8. Consider including environmental, educational and retail outcomes associated with vapes in modelling***

 Within our workshop, these outcomes of vape policies were considered to be important; however, the focus of this project was on the health outcomes. Further research should be undertaken around the evidence available for modelling environment and retail outcomes for vape policy modelling.

***8.3.9 Invest in the development of individual-level health economic modelling for a UK population to assess new vaping policies***

We recommend that an individual-level health economic model be developed for a UK population which can estimate the long-term impacts of vape policy options. Stakeholders should agree the scope of the model, firstly to ensure that appropriate age ranges are included within the model (so that both the harms of vapes for young people who have never smoked can be included as well as the benefits for individuals quitting tobacco smoking using vapes), secondly to ensure that important subgroups of the population are included (e.g., those within mental health conditions and those with low socioeconomic status), and thirdly to ensure that sufficiently broad model outcomes are included (e.g., environmental outcomes and retail outcomes).

***8.3.10 Develop a modelling platform to flexibly assess comprehensive policy options***

Investment in vape modelling risks being undermined by developing models that differ, perhaps unnecessarily, in their mechanistic structure and use of data without this being well documented. To address this, there is a need to apply the FAIR principles—Findability, Accessibility, Interoperability, and Reusability (further detailed in 8.4)—to both data and code in vape modelling ^183^.

As modelling in this space becomes more specialised—tailored to different interventions and policy options and to particular population groups—it also becomes more diverse in its mechanisms and use of data. This growing complexity highlights the limitations of standalone models, which often lack flexibility and cannot easily and quickly be adapted or reused across different contexts. A shift toward a platform-based approach is needed.

A modelling platform offers a structured yet flexible foundation that could support a range of modelling approaches while promoting consistency across these approaches where this is appropriate. Inspired by platform trials in clinical research ^184^, this approach could involve building shared infrastructure—tools, methods, and processes—that can be applied across modelling projects, e.g., some of the model mechanisms and data required for modelling vape tax changes will be needed for modelling flavour restrictions, but not all. Instead of aiming to build a single model suited to all the policy questions in vape research, a platform approach would support the development of multiple, purpose-specific models within a coherent, interoperable framework.

To be sustainable and adaptable, the platform should be developed on sound software engineering and open science principles. This includes using modular code, version control, documentation, ontologies for variable definitions, and testing, while ensuring transparency and open access wherever possible. These practices will not only encourage wider uptake but also improve the credibility of the modelling by enabling peer review and replication. Furthermore, a platform approach could also help to coordinate the efficient and consistent use of existing data, e.g., through shared access and shared data processing code, and to guide plans for the collection of new data.

### 8.4 Recommendations for public sharing and communication among research teams working on vape data collection and analysis

The task before us is to make better use of the existing datasets and to fill the gaps in evidence with new data collection. This work cannot be done within a single project; coordination across projects is needed to help ensure a coherence in the evidence-base to inform vape policy in the UK. This need for coordination covers the design of new data collection, the sharing of existing data, and the code used to analyse these data. This should also follow guiding principles to follow best scientific practices (e.g., by collaborating with the UK reproducibility network https://www.ukrn.org/).

It is understandable to think that large-scale coordination across projects is difficult to achieve. However, there are ample examples of national coordination among research teams as happened in the UK Centre for Tobacco Control Studies (2008–2013) and UK Centre for Tobacco and Alcohol Studies (2013–2018). The key principle behind the success of these centres was that they increased communication and understanding among research groups around the country, and out of this came coordination, coherence and collaboration. Collaboration between modellers and behavioural scientists is important in developing useful models ^19^.

A new national structure should be established to facilitate this communication and understanding among research groups working on tobacco, vaping and other nicotine-containing products. Ideally, this would also involve integration with government departments and other non-research stakeholders to increase the flow of people across the research-policy-civil society interface (e.g., by building in CASE-style fellowships). However, in the absence of a formal structure there are still key principles that can be followed to reach the same goal. Key among these are the FAIR data principles ^183^, which can also be applied to the code used to process and analyse data, as outlined below.

***Findable***

Building on the review of data sources presented in this article, it is recommended that a resource is created to make the available UK data for vapes more easily discoverable, including developing full and coherent metadata and persistent identifiers to allow different data sources to be cited and for those citations to be tracked.

***Accessible***

The data sources needed to inform the effects of specific policy options are diverse and can often be inaccessible. There are two key challenges. First, key market research data can be very expensive to purchase and when purchased by one research group may not be accessible by another research group without a further purchase. A solution would be for these data to be purchased centrally and made equally accessible to research groups nationally, following a UKCTAS-type arrangement. Second, data sources can be owned by specific agencies, regional organisations or local authorities. These sources are often in the form of routine service monitoring data and could be further “unlocked” to help them to inform national decision-making ^185^.

***Interoperable***

Without a watchful eye, new data collection risks introducing unnecessary variation to the definitions of key variables, making data sources harder to combine and integrate. A solution would be to develop a Community of Practice (CoP) for new vape data collection, which shares and reviews protocols. In addition, the CoP could promote the use of ontologies to help ensure the consistent definition of key variables.

***Reusable***

The research community is now routinely making code as well as data open source, with appropriate usage licenses and version control. An easy way to make data more reusable for modelling across different modelling projects is to share the code used to process that data to produce model inputs. This sharing can be achieved using online repositories such as Github, with version control and data citations used to associate specific versions of code with specific versions of data.

***PPI engagement***

To maximize the value of PPI in modelling studies, researchers should establish diverse stakeholder panels at the outset, including variation in socio-demographic characteristics and nicotine product use patterns (e.g., current vapers, dual users, former vapers). Following the PACTS principles, panel members should be actively engaged throughout all stages of modelling work, from initial design through result interpretation and dissemination. This collaborative approach requires systematic documentation of PPI contributions and adequate resources, including upfront training for panel members. Researchers should also establish mechanisms for ongoing feedback and appropriate compensation, creating sustainable partnerships that maintain transparency about how public input shapes model assumptions and policy recommendations ^186, 187^. PPIE feedback highlighted the need for sustained engagement to ensure that “evolving policies remain grounded in real-world experience and continue to reflect public priorities”.

### 8.5 International views on recommendations and generalisability

Given the heterogeneity of meta-analyses (e.g., in terms of methodology/definitions of vaping), one international expert thought that effect sizes from reviews should be treated with caution. Another expert felt that intrinsic risks of nicotine use *per se* should be acknowledged. Given the lack of long-term data on vape use, it was suggested that evidence on the effects on cardiovascular disease and cancer could be drawn from looking at snus use, common in Scandinavia, which generally shows detrimental effects are limited ^177, 178^. Another harm-related issue for nicotine use, not addressed directly, was addiction itself. In terms of effects of potential policy effects, missing evidence (now included) was identified on cross-price elasticity and additional evidence in relation to flavour restrictions pointed out. This generally indicates that while this reduces vape use, an unintended consequence is that restrictions may increase cigarette sales ^188– 190^.

Several experts commented on the modelling approach proposed. A preference was expressed for using quasi-experiments or natural experiments over experimental tobacco marketplace approach to parameterise potential policy effects (together with elicitation from experts). It was argued that this would lead to more generalisable findings, which would not necessitate separate consideration of industry responses as this would be incorporated in this natural setting. We suggest that a triangulation of methods may be the best approach. However, it is also important to note that there are few precedents to many of the vape policy options now being considered for the UK, and no precedents in the UK context. Therefore, whilst evidence from past policy changes may be preferable, behavioural economic experimental evidence may in some cases the best option to inform policy decisions. Furthermore, given the rapidly changing nature of the vaping (and wider nicotine) market, experimental evidence may be required to generate timely evidence for new policy scenarios.

Experts also felt that intersectionality should be considered when assessing impact on priority groups and that the role of other products growing in popularity (especially pouches) needs to be incorporated into any model. Some experts thought that vapes should be embedded with behavioural support in the modelling, as vape use alone is less effective, which suggest that our Person theme should have a higher priority for modelling. There was some agreement that any model must not become more complex than it needs to be, as this likely will introduce uncertainty (making it more difficult to identify biases) thus could cause models to perform worse, making effects hard to estimate reliably. It was also suggested to use tobacco policy effects to estimate vaping policy effects (where this is available), but it is important to maintain the distinction between tobacco use and vaping as there are large differences in the nature of the behaviours, the associated harms, and the policy approaches to each behaviour may have different goals. Using multiple models and different approaches to see how they compare could get at some of the uncertainty in effect estimates.

Another issue raised was the treatment of dual use in any model. Experts agreed that dual users are a very mixed group, some of whom may have quit smoking if it had not been for vaping but for others it may have helped them transition out of smoking. Data from the US PATH could be interrogated to further categorise this heterogenous group ^191, 192^.

A key point that was made by all international experts was the great divergence between countries in public health messaging, regulatory approach to vaping and likely consequent use of vapes, meaning that evidence from other countries should be used cautiously. For instance, while some countries focus on achieving a tobacco-free future, others (notably Australia, New Zealand and the Netherlands) are working towards a nicotine-free world. The UK is seen to have a more permissible attitude towards vapes, which has resulted in wide adoption of vapes as a smoking cessation aid. By contrast, in other countries vapes are portrayed as worse than smoking in the media (e.g., Australia), use of vapes has remained relatively low (e.g., in Netherlands), dual use rates are very high (e.g., Netherlands, Italy) with other products being equally as popular (e.g., nicotine pouches and heated tobacco products in Switzerland/Italy). Some experts felt that there was good evidence for gateway effects of vaping into smoking due to cigarettes being relatively cheap (e.g., in Italy) and that use of unregulated vapes was particularly high in younger people (e.g., Netherlands). The upshot of this is that UK-specific modelling will likely only have limited generalisability to other jurisdictions, given very different use patterns, regulatory approaches and general public attitudes. The observation of important between-country differences was also reflected by stakeholders from different UK nations (Wales and Scotland), who thought the article needs to reflect differences in vaping between the UK nations. As an example, Scottish colleagues pointed out that Scotland does not provide vapes as part of stop smoking services or have a Swap to Stop scheme and in Scotland, it's not clear vaping leads to smoking cessation and vapes are not the most impactful cessation aid there. Other relevant differences include that Scotland has a registration system for tobacco and vape retailers and a child can currently be criminalized for vaping (though this should change with the Tobacco and Vapes Bill).

## 9. Discussion and conclusions

This data mapping project sought to establish what type of research about vapes would be most useful for modelling of priority policies in the UK context, what data already exist to go into this type of analysis and what new data are needed. The project used a mixture of methods, including stakeholder input gathered through in-person and online workshops, bespoke evidence and data searches and conceptual modelling, all underpinned by PPIE input. The workshops involved key national/local policy makers, non-governmental vape policy experts, lay members, national and international experts in public health and behavioural science, commercial determinants of health, data collection/analysis and modelling methods. The key three policies agreed on by key stakeholders to focus on were ‘Price’ policies (e.g., taxation of vapes), ‘Prescriptive’ policies (e.g., restriction on marketing but also on products, such as banning certain vaping products), and ‘Place’ policies (e.g., where one can vape or buy them). Key target groups identified were young people, people experiencing socioeconomic disadvantage and people who smoke. Key outcomes - in addition to vaping, smoking and nicotine use outcomes - were health inequalities and health impacts. Behavioural systems mapping for each of the top three policies identified factors related to sociodemographic and socioeconomic characteristics (e.g., age, SES), behaviours (regular vaping/smoking, use of unregulated vapes) and behaviour-related harms (both tobacco and vaping-related harms), lifestyle and psychological factors (e.g., norms, dependence) that applied to all three priority policies, with some policy-specific differences in the maps.

Given that the developed behavioural systems maps highlighted many interacting factors, dynamic, non-linear, individual-level complex systems models were judged to be most appropriate to capture the impact of prioritised policies within a given social and industry context. No such models were found to have been developed in the UK context. However, we recommend starting with a simpler model and gradually building in this complexity due to gaps in current data.

The essential evidence required to parameterise such a model would be on 1) transitions between vaping and smoking and their interaction (across key subgroups); 2) the relative harms associated with vaping compared with smoking and no product use; and 3) how policies affect transition between various smoking and vaping states.

Given these requirements, we looked at key reports of prior evidence, which suggested 1) vaping is helpful for people who smoke trying to quit, but evidence on whether or not vaping among people who have never smoked causes later smoking is less clear and 2) vaping is less harmful than smoking but evidence on absolute health effects compared with no product use was relatively limited and 3) no systematic review on policy effects in the UK had been undertaken.

In order to fill these evidence gaps, rapid reviews were undertaken. These found that there is already substantial evidence on transition probabilities between different vaping and smoking states in the UK, including for some priority subgroups (but less so for those from more disadvantaged socioeconomic groups and those with mental health conditions). By contrast, there was very limited UK evidence on the health effects of vaping versus not using anything. Evidence of policy impacts on transition probabilities was very limited in the UK. Finally, there was some evidence internationally that restrictions (e.g., on particular products or flavours) are being circumvented by industry.

Given the need to parameterise a complex system model, we sought to identify existing data sources which may fill the data gaps identified above. This yielded a relatively large number of primary data sources representative of the UK, providing good coverage on relevant key characteristics (including vaping and tobacco use prevalence; sociodemographic characteristics) across both cross-sectional and longitudinal datasets. However, less information was generally available about vaping-related than smoking-related characteristics (such as vaping quit attempts and motivation to quit). A number of market data sources relevant to the UK were also found, which could largely cover the priority policies under consideration in this article. In addition, data were very fragmented, undermining easy consolidation of information in one place.

To supplement UK-relevant information, where there was limited evidence, we sought input from international stakeholders on non-UK evidence that could be used instead to support the development of a policy model. Non-UK evidence on vape harms suggests that people who vape are exposed to significantly fewer harmful chemicals, carcinogens and toxicants than people who smoke cigarettes but to more harmful substances than non-users. The evidence also suggests that at the individual level prior vaping is associated with later smoking in young people but that causality is unclear. Population level evidence is generally consistent with the idea that increases in vaping over time are associated with decreasing cigarette consumption at the aggregate level, including among youth (indicative of diverting people that would be smokers away from starting to smoke). However, none of the evidence – whether at individual nor ecological level – is of sufficient quality to allow strong conclusions to be drawn. In terms of policy effects, there is a consistent picture emerging on vape taxation, based mainly on US evidence. Increases in vape taxation result in vape use reduction. There is, however, some evidence that this price-instigated reduction in vape use may also result in concomitant increases in cigarette use.

International experts provided feedback on the evidence and modelling approach for assessing vaping harms and policy impacts. Some felt important reviews were initially missed, particularly regarding vaping harms, addiction, and cross-price elasticity of demand between vaping products and tobacco, which were subsequently included. Concerns were raised about the heterogeneity in meta-analyses and limitations in long-term data, with suggestions to draw on snus studies for cardiovascular and cancer risks. Experts preferred using natural or quasi-experimental data over experimental marketplace approaches where available, arguing this would enhance generalisability and better account for industry responses. The modelling of dual use was flagged as critical due to the heterogeneity of this group, with US PATH data recommended to refine classifications. Experts also highlighted the importance of considering intersectionality, emerging products (e.g. nicotine pouches), and embedding behavioural support in models. They cautioned against overcomplicating models, as complexity could increase uncertainty. This is in keeping with our recommendation to develop an initial core individual-level model, and then to gradually add complexity. The value of having undertaken a systematic model planning and data mapping exercise as in this article is that it allows decisions to be made on when simpler models might be more appropriate, and also what key effects might be missed when using a simple model.

A consistent theme was the divergence in international regulatory contexts and public attitudes toward vaping. Similar concerns were expressed by stakeholders from different UK nations, highlighting differences in the policy landscape and approach to vapes. The UK’s more permissive stance contrasts sharply with more restrictive environments like Australia and the Netherlands, where vaping is less common, dual use is higher, and with Switzerland where alternatives like heated tobacco are more prevalent. As a result, it will be important to incorporate uncertainty around use of any non-UK evidence to reflect the different contexts, and UK-specific models may have limited relevance for other countries.

Based on the data mapping exercise and the likely data requirements for a policy model of sufficient complexity, we recommend some additional primary research in the UK. First, longitudinal studies that include more detailed information on vaping behaviours and the factors influencing vaping are needed, which undertake frequent enough data collection to allow estimation of transitions between behaviours in response to policies being implemented. This could be achieved by adding new questions to existing data sources, though the complexity of question required may make this difficult to achieve. Second, given the current lack of quasi-experimental evidence, experimental studies (either in the lab or nested in population surveys) are needed to estimate hypothetical or actual impacts of policy on product purchasing. Third, more information on health impacts (in particular of vaping vs not using any product among people who have never smoked and of use of regulated vs unregulated vapes) is needed. This could be achieved by linking detailed behavioural characteristics in existing data sources (see point 1. above) to health records and mortality datasets ^193^. Fourth, in order to get a better understanding of the size of the unregulated vape market, HMRC could in the future use data on taxed vaping products and the overall vaping market to estimate the scale of the illicit market (as it currently does for tobacco). In addition, focus groups could be run to test survey questions for assessing the illegal vape market, such as the type of products available and sources of purchase. Finally, within surveys measures should be standardised, including consistent definitions for ‘regular use’ and ‘ever use’ of smoking and vaping. Local authority level school-based questions on health-related behaviours should be standardised and, ideally, collated at a national level.

Taken together, there are several implications for modelling vape policies. As existing UK modelling studies have used incomplete transition diagrams that fail to fully capture the interactions between vaping and smoking behaviours despite the availability of good evidence on individual transition probabilities, it is recommended to calibrate a coherent system of age-specific transition probabilities using these existing estimates and survey data, accounting for time trends and key subgroups such as those from more disadvantaged socioeconomic groups or with mental health conditions. Further, given that the priority policies (price, prescriptive and place) encompass a variety of actual policy options, a complex systems approach could support understanding on how these options interact to affect behaviour, informing policy logic model development and any subsequent evaluation. However, due to limited UK-specific evidence on the effects of past policy changes, it is recommended, where feasible, to model policy impacts using mechanistic structures that link each step of behavioural change to the best available evidence and test key assumptions. While common components like life course dynamics can be informed by existing UK data, there is a critical need to generate new evidence on how UK consumers might respond to specific vape policies such as pricing, flavour bans, or retail restrictions. Building mechanistic models that break policy effects into a series of steps helps us to be explicit about how industry might act to modify the effects of policy changes. These models also enable detailed estimates of how consumers may adjust their consumption across multiple products, accounting for variation across socio-economic groups. It is important to link mechanistic models to evidence from quasi- or natural experiments where possible, for example through calibration or external validation. However, we must also recognise that past observations may not reflect current market dynamics or consumer vaping behaviour. A further advantage of mechanistic models is that they allow us to incorporate experimental evidence, particularly from behavioural economic studies, to produce detailed predictions of consumer responses. Thus, when direct evidence is unavailable or not representative of the current context, mechanistic models combined with behavioural economic evidence and an exploration of the effects of key assumptions may be the best available option.

For specific inputs, the key lack of useful data concerns the absolute harms of vaping in people who have never smoked (and transitions from vaping to smoking), which requires *de novo* data collection. In addition, mechanisms of actions likely vary across policies and will therefore require specific analysis (e.g., for place-based interventions spatial analysis could be incorporated within agent-based models). Due to the frequent and impactful ways industry has circumvented vape regulations—such as through new products that may mitigate restrictions on flavours (e.g., through accessories) or nicotine content (e.g., through increasing bioavailability) —policy models should incorporate or at least acknowledge potential industry responses. They should also capture unregulated vape use as this may carry greater risk than regulated vape use, in particular for vapes containing illicit/banned substances (and increase in response to policy changes) as well as interactions with other nicotine products. Environmental and retail outcomes were considered outside the scope of this project, but we recommend that an individual-level health economic model be developed for a UK population to estimate long-term impact of vape options incorporating those outcomes as well as health outcomes across appropriate age ranges and important subgroups.

In producing this article, it is important to acknowledge PPIE involvement, which enhanced the study’s relevance, ethical robustness, and potential policy impact. The consistent engagement of a diverse group enabled deeper contextual insight and improved the accessibility and credibility of outputs. This is reflected in feedback received by PPIE members who commented that they “appreciated the openness of the research team to genuinely considering PPIE feedback, not only during the workshops but also through follow-up opportunities”. Clear communication was praised throughout as an enabling factor to engage with a complex and often technical topic. While socio-economic diversity could not be formally assessed due to the sensitive nature of the topic, the approach fostered trust, continuity, and meaningful integration of public perspectives into the research process. One key insight provided by PPIE (reinforced by other stakeholders) was the importance of considering harm perceptions in driving behaviour. Given representations by the media but also by health professionals that vapes may be as or more harmful than smoking, this could mean that vape policies may reinforce such misperceptions, thus undermining the use of vapes for smoking cessation by smokers.

Finally, given the required complexity of the modelling and likely limits on resources, we also feel it is important to make two broader recommendations. First, to ensure the credibility and usefulness of vape modelling, it is essential to avoid unnecessary divergence in model structures and data use by applying FAIR principles and promoting transparency. As models become more complex and tailored, a shift from standalone models to a platform-based approach—built on open science and sound software engineering—can support consistency, adaptability, and reusability across projects. This would enable shared tools, methods, and data resources, fostering collaboration and improving the efficiency and reliability of policy modelling in this field. Second, and relatedly, as a comprehensive model to evaluate the impact of potential vape policies requires input across many different disciplines, including but not limited to behavioural and medical sciences, mathematical modelling, epidemiology and health economics, requiring coordination across different projects, it is recommended that a new national structure (akin to past structures such as the UK Centre for Tobacco Control Studies) is established to help ensure a coherence in the evidence-base to inform vape policy in the UK.

In conclusion, this data mapping project highlights both the opportunities and critical gaps in developing robust, UK-specific models to assess the impact of vape policies. Addressing these gaps will require targeted new research—particularly on vaping transitions, harms among people who have never smoked, and industry circumvention—as well as the adoption of complex, flexible modelling approaches grounded in the FAIR principles and supported by a coordinated national research infrastructure. By fostering collaboration across disciplines and ensuring transparency and consistency in modelling efforts, the UK can build a credible, evidence-based foundation for shaping effective vape regulation.

## Data Availability

All included data used in this manuscript as well as extended data can be found at the Open Science Framework repository: https://osf.io/8zaxc/ titled SPIRE Project; DOI: https://doi.org/10.17605/OSF.IO/8ZAXC ^194^ *Data are available under CC-By Attribution 4.0 International*
