# Supplementary material for: Scoping of Policy Impacts for Regulating E-cigarettes (SPIRE): findings from a data and decision analytic model mapping project
Source: NIHR Open Res. 2025 Oct 6;5:92. [Version 1] doi: 10.3310/nihropenres.14038.1 (PMC12805243; doi:10.3310/nihropenres.14038.1)
Supplement: Peer review history

# Reviewer report: Reviewer response for version 1

**DOI:** 10.3310/nihropenres.15267.r38307

**Published:** 2026-01-14

**Authors:** Donald Kenkel

**Affiliations:** 1 Cornell University, Ithaca, NY, USA

This paper is a scoping review of data sources and gaps in the data required to develop modeling studies of vape policies to help guide regulating e-cigarettes in the UK. Population health simulation models are a very useful approach to synthesize evidence and make predictions about the impacts of vape and tobacco control policies. Mendez and Warner (reference 35 in the paper) use a simulation model to highlight the potential for vaping to increase smoking cessation and reduce life-years lost due to smoking. Although they find positive policy impacts under a variety of scenarios, the wide range of estimates also highlights policy uncertainty. In a UK-specific study, Levy et al. (reference 13 in the paper) use an indirect simulation method to estimate that from 2012 - 2019, the use of vapes helped decrease smoking rates and averted an estimated 166,000 smoking-related deaths. In official regulatory impact analyses, the US Food and Drug Administration used population health models to estimate the policy impacts of proposed tobacco product standards to prohibit menthol cigarettes and to limit the nicotine yield of combustible cigarettes (FDA 2022, 2025). This paper makes important contributions to the development and use of similar population health models for UK e-cigarette regulatory analysis.

 The authors took a systematic approach that included stakeholder engagement, identification of existing UK datasets, and literature and evidence reviews to develop recommendations for modeling e-cigarette regulations. Beyond contributions to policy modeling, many steps in the analysis will be of broader interest for vape policy researchers. For example, the data dictionary, which will be a "living document" that can be updated with new information, is a valuable resource for researchers to understand what data are currently available. The recommendations for future work provide an agenda for conducting research that will fill important gaps in the evidence-base for vape regulation. 

 The authors state that Patient and Public Involvement was a core component in gathering stakeholder input. In this light, it is surprising that the list of stakeholders does not explicitly include smoking cessation patients, or smoking and vaping consumers more generally. Many of the stakeholders in the workshops were policy makers, policy enforcers, and policy advocates, together with academic researchers. The paper does not discuss whether any of the workshop participants were current or former vapers or smokers. The lived experience of the consumers of the regulated industry could provide important insights beyond the experiences of the regulators and academics. More controversially, an argument could be made to include vape manufacturers and retailers as important stakeholders. US federal agencies are required to conduct impact analysis of major regulations; the federal agencies and the Office of Information and Regulatory Affairs routinely gather input from consumers and the regulated industries.

 Stakeholders were asked to rank six policy themes, which resulted in the identification of three priority themes of Price, Prescriptive, and Place. Most of the policy themes were framed as steps to reduce vaping, such as higher vape taxes to increase price, bans of certain vape products and flavors, and restrictions on where people can vape or buy vapes. Notably, the paper reports that the majority of workshop participants suggested that many of these policies might decrease quit attempts and increase smoking. Only one of the six policy themes focused on strengthening support for smokers using vapes to help them quit or cut down; this policy theme was not included in the final top three policy priorities to be modeled.  A different framing of the policy themes or a broader set of stakeholders might have placed greater emphasis on the potential public health gains from vaping to help smokers to quit, especially socioeconomically disadvantaged smokers and those with mental health conditions.   

 The team conducted careful and thoughtful literature reviews on three key questions -- the health and well-being impact of vaping on never-smokers, the transition probabilities between smoking and vaping the impacts of the priority policies on these transitions, and industry responses to government policies. On the first key question, the review found very little UK-specific evidence on the health and well-being impact of vaping on never-smokers. However, the lack of UK-specific evidence is probably not too important for the modeling exercise. Vapes do not expose users to the combustion-generated toxicants in tobacco smoke that are linked to the major smoking-related causes of death and illness. The much lower levels of toxicants found in vapor, the much lower levels of biomarkers of exposure, and population-level evidence such as the Swedish experience with snus makes a compelling case that the health risks of vaping are very low regardless of the nationality of the users.

 On the second key question, the review also found limited evidence on the transition probabilities between vaping and smoking and especially on the impacts of policies on these transitions. The answers to these social science research questions are likely to vary across countries, which poses a challenge for developing UK-specific estimates. At the same time, the experience of other countries also provides the opportunity to make stronger causal inferences about the impacts of policies on transitions. For example, US states have adopted different tax rates and different policies towards flavored vapes, which provides quasi-experimental evidence on policy impacts. US research is building a body of evidence not only on the price-elasticity of vapes, but also on the extent to which vape taxes and flavor bans cause consumers to substitute into combustible cigarettes. With care, this evidence can be extrapolated to inform estimates of transition probabilities that are needed for UK-specific public health simulation models. 

 On the third key question, the review again found limited UK-specific evidence on industry responses to vape policy. The review of international evidence reviews whether a disparate set of industry responses "undermine" vape policies. Responses like selling and promoting banned products are illegal and may blunt the policy's intended consequence to reduce use (Kenkel et al. 2025).  Other responses, like passing-through tax hikes to consumer prices or product innovations, are predictable implications of profit-maximization which should be taken into account when developing policies. And still other responses, like lobbying and arguing against the policy, are part of the democratic process of regulation in a market-based economy.

  References Cited

 Food and Drug Administration (2022). Tobacco Product Standard for Menthol in Cigarettes: Preliminary Regulatory Impact Analysis, Initial Regulatory Flexibility Analysis, Unfunded Mandates Reform Act Analysis. Silver Springs, Maryland: U.S. Food and Drug Administration. https://www.fda.gov/about‐fda/economic‐impact‐analyses‐fda‐regulations/

 tobacco‐product‐standard‐menthol‐cigarettes‐proposed‐rule‐preliminary‐regulatory‐impact‐analysis.

 Food and Drug Administration (2025). Methodological Approach to Modeling the Potential Impact of a Nicotine Product Standard on Tobacco Use, Morbidity, and Mortality in the U.S. Technical report, HHS, FDA, Center for Tobacco Products, Silver Spring, MD.

 Kenkel, Donald, Alan Mathios, Grace Phillips, Revathy Suryanarayana, Hua Wang, and Sen Zeng (2025). “Understanding the Demand-Side of an Illegal Market: Prohibition of Menthol Cigarettes.” Health Economics 34 (5): 956-971.

Is the work clearly and accurately presented and does it cite the current literature?

Yes

If applicable, is the statistical analysis and its interpretation appropriate?

Not applicable

Are all the source data underlying the results available to ensure full reproducibility?

Yes

Is the study design appropriate and is the work technically sound?

Yes

Are the conclusions drawn adequately supported by the results?

Partly

Are sufficient details of methods and analysis provided to allow replication by others?

Yes

Reviewer Expertise:

economics, regulatory impact analysis

I confirm that I have read this submission and believe that I have an appropriate level of expertise to confirm that it is of an acceptable scientific standard.

## References

[1] : Understanding the Demand-Side of an Illegal Market: Prohibition of Menthol Cigarettes.2024; 10.3386/w32148 10.3386/w32148 39967408

# Reviewer report: Reviewer response for version 1

**DOI:** 10.3310/nihropenres.15267.r38313

**Published:** 2025-12-31

**Authors:** Ian Irvine

**Affiliations:** 1 Concordia University, Montreal, Canada

NIHR Open Research. Oct 06, 2025.

 “Scoping of Policy Impacts for Regulating E-Cigarettes (SPIRE): findings from a data and decision analytic model mapping”, by Squires, Gillespie, Kock et al. A comment.

 Ian Irvine, Professor, Economics, Concordia University Montreal[1]. Ian.irvine@concordia .ca

**Introduction:** The Squires et al review paper (henceforth Review) is a detailed and comprehensive presentation of the elements pertaining to the regulation of vaping and to the construction of a multi-purpose open model on nicotine-use behavior. Even readers well-versed in the area will not fail to learn from this contribution. Its content is decidedly in keeping with a harm-reduction philosophy and how new products can contribute to an improvement in health. My main focus here  is upon how to address the contentious issue of whether to incorporate evidence and information from the supply side of the nicotine and tobacco market. I favor evaluating all available information and filtering out less useful information *ex post* rather than filtering out all information from specific sources *ex ante*.

**1. The Objective Function**. The pursuit of better nicotine health is the objective of the paper, and policy measures need to be tailored to this objective. To this end, fuller information is required, both in terms of usable data and upon how humans react to incentives. Economists portray the attainment and formulation of goals as the outcome of a production process. The inputs are data, logic, incentives etc. pertaining to all of the products that impact nicotine health. Policies impact the consumption of both e-cigarettes and combustibles.  In this context, good vaping policy should simultaneously involve good combustibles policy, and policy initially directed towards cigarettes may complement e-cigarette policy. To be specific: given that e-cigs are perhaps an order of magnitude less toxic than combustible cigarettes, could we think of policy that might incentivize switching that is initially directed to smoking rather than vaping? To illustrate:

 Could negative warning messages on both sides of a cigarette pack be re-constructed so as to contain positive escape advice at least on one side? Would it be possible to inform smokers about less toxic substitute products on the face of a cigarette pack? In this case, ‘good’ vaping policy could see an increase in vaping as a result of an off-ramp attack on an adjoining deathly product with a similar pharmaco-kinetic profile.

**2. Incentive-compatible policy and the traditional model**. The standard model of regulation is one in which there is a beneficent regulator who regulates the market in the interests of the population, large parts of which are nicotine dependent and poorly informed on the consequences of the different modes of ingesting nicotine. In this model the supply side is generally portrayed as an antagonist, seeking to upend regulations that may reduce profits, and the demand side is composed of individuals who need guidance and chaperoning in attaining better health. The main  antagonist, Big Tobacco, has a history of being untruthful. It is painted in the current era as jumping on the harm-reduction bandwagon because it needs to keep shareholders content while simultaneously recognizing the strong social pressure to reduce its sale of life-terminating combustible products. Big Tobacco supplies about one quarter of the e-cigarette market in Canada. It is not obvious if the Review wishes to keep all of the supply side research and influencing away from policy or just Big Tobacco---which undertakes much of the pro harm-reduction research.

 Incentive compatible policies could be explored: rather than having the supply side as a constant  adversary, could we devise measures that suppliers may not have an incentive to sidestep? Are there policies that are in the interest of the regulator (and *sic* citizens) that might be simultaneously in the interest of the supply side, or at least not contrary to its commercial interests? The answer may be yes: all major cigarette producers claim they intend to be selling “mostly smoke-free products” by a specific date. While one can entertain doubt about such claims, it may be possible to test their sincerity---by posting positive switch messages on packs for example, as described above. If impactful, such messages would benefit the supply side and the demand side simultaneously: they would improve health without causing much financial damage to the balance sheets of suppliers. Such damage is not an objective here, it is a constraint in the optimization. The Swap to Stop campaign is incentive compatible.

 This thinking appears antithetical to the Review (see section 4.1). The Review states that (a) its stakeholders are, and should be, independent of industry and (b) that industry frequently raises concerns around illicit product use to stymie incoming regulation. *Ergo*, industry-based information should be excluded. As for (a), should the study of nicotine markets exclude the considerable stck of knowledge embedded in e-cigarette supply that is independent of traditional tobacco companies? As for (b), is it unreasonable for a legal entity to discuss the illegal market? The Review states that industry interferes with policy making and implies that industry’s warnings about the illegal market effects of regulatory policies are disingenuous. Evidence indicates that 80% of the disposables market is illegal in the US because the products have not received FDA approval. In Canada the illegal side of the cigarette market produces about half as much as the legal market. In such circumstances an effort by legal producers to warn about illegality seems reasonable. The Review implies otherwise.

**3. Research from industry**. Industry studies are excluded from the Review (section 2.3.2) to avoid bias because Big Tobacco is not trustworthy in its research.

 Modern Big Tobacco has become a bifurcated entity, and perhaps the name Big Tobacco should be replaced with the term Big Nicotine.  On the one hand it continues to push life-shortening products,  and on the other hand it spends hundreds of millions of dollars annually in the development and marketing of new lower-risk products---products which, paradoxically, can save users from the effects of the traditional side of their business. The lower risk products component of the business is much like the legal alcohol or legal cannabis or legal gambling businesses. It supplies products which do not improve health but that are accepted as products that individuals are free to use in a modern society. It is where the research originates and is the source of papers and presentations to scientific conferences and journals that generally do not fall within the orbit of health conferences. But the papers are accepted and evaluated on the basis of their content. Health conferences frequently shun these papers and equally may prevent their authors from even attending health conferences.

 Research produced by industry may be equally careful as research from the academy: industry-sponsored authors know that their papers will be heavily scrutinized and if the authors/researchers err they may be fired. In contrast, errors made in the ivory tower are forgivable---the researchers may have tenure and cannot be easily dispensed with.

 An alternative view of industry research is that readers know the predisposition of the researchers and therefore the research output is not worth reading. The same argument can be made about researchers who are funded by Global Action to End Smoking on account of bias favoring harm reduction. Equally, papers produced in institutes or schools funded by Bloomberg are less likely to be favorable to harm reduction. Where do we draw the line?

 Up-front exclusion, whether it be to exclude papers or individuals, risks creating a culture of elitism.  The Review proposes that the way forward is to accumulate better data, develop more surveys, perform additional statistical work, engage in better model building while communicating with similarly minded colleagues.

 How many authors of spurious ‘metals’ studies have ever visited a vape shop and asked the high-school dropout behind the counter about a dry hit?

**4. Talking with the supply side**. I recently completed a series of 15 extended semi-structured interviews with a wide variety of workers, owners and suppliers in the non-combustibles market in Canada---we excluded Big Tobacco from these interviews, ostensibly to avoid bias, but in reality to gain acceptability with the academy. This undertaking led to a greatly improved understanding of the market and human behavior. The examples are too numerous to elucidate in a brief review. The work of researchers with PhD credentials would only be strengthened with the knowledge available from successful entrepreneurs.

**5. The model**. I was unsure about the nature of what the envisaged model might produce as output. I appreciate that it is intended to be multi-functional and open. This would be very positive. Will it be a dynamic model that will reach a steady state in n years, based upon state-transition probabilities that can be influenced by policy? Is the envisaged time horizon important; or will it produce two steady states---now and the future, without concern about time? Will discounting be necessary? Biostatistical models frequently estimate the costs and benefits in terms of life years saved and the monetary values of these years are discounted back to a base period. The discount rate is critical in these models because saved life years usually occur very far into the future, and, unless discounted by a very low discount rate, those saved years can become almost inconsequential. In Canada, federal government researchers are required to use a high rate, specified by the Treasury Board, and this makes modeling in this area quite puzzling, because it ultimately puts little value on life years saved, when discounted. I believe that the US now uses a discount rate of 2%. To take an analogy: if the Greenland ice sheet takes 200 years to melt, the ensuing cost is minimal if discounted back to today at high discount rates, even if it may cause catastrophe down the road.

**6. A new national structure**: The very end of the Review promotes the founding of a type of national research centre or structure, though what resources would be required for this is not explored. The envisaged structure might be just in the form of increased liaison between researchers with some minimal financial support, or it might be something bigger. If it is the latter that the Review envisages, the idea might have been fleshed out.

 If such an agency or structure were to be formed, an important question is whether the ultimate model that might be produced would be open to all researchers, regardless of where they work.

**To conclude**: The Review presents a detailed overview of the state of academic knowledge and poses many of the important questions that require answers. Simultaneously, the decision to amputate the supply side of the market as a source of information poses a paradox. Industry supplies devices that can be used either as a utility-enhancing source of nicotine or a quit-smoking device. Some of the authors of the Review are associated with researching and promoting this fact. Their work complements the countless Cochrane reviews and monographs from the Royal College of Physicians. Their decision to dismiss the views of the producers who supply these same products as a source of information poses a conundrum: in the UK, the number of vaping device users approximates the number of people who smoke. The people are speaking, and they are putting their trust in the supply side---where the Review declines to venture.

 The use of the word industry in the Review suggests a relatively homogeneous and untrustworthy group. In reality, it is multidimensional. Just as the authors are capable of distinguishing bad research that passes their filter from good research, they are equally capable of doing the same for those papers that are automatically filtered out *ex ante* on account of their provenance.

 [1] **Disclosure**: I appreciate being asked to review this paper. I am a professor in economics at Concordia University, Montreal. I have worked as a consultant to the federal government of Canada on alcohol and tobacco policy; I have worked for lawyers in the private sector; some recent research of mine has been funded by Global Action to End Smoking and I have accepted conference costs from nicotine producers. I speak with as many people as possible in the nicotine sector, whether they be health advocates and regulators or suppliers.

Is the work clearly and accurately presented and does it cite the current literature?

Yes

If applicable, is the statistical analysis and its interpretation appropriate?

Yes

Are all the source data underlying the results available to ensure full reproducibility?

Yes

Is the study design appropriate and is the work technically sound?

Yes

Are the conclusions drawn adequately supported by the results?

Yes

Are sufficient details of methods and analysis provided to allow replication by others?

Yes

Reviewer Expertise:

The economics of public policy

I confirm that I have read this submission and believe that I have an appropriate level of expertise to confirm that it is of an acceptable scientific standard.

# Reviewer report: Reviewer response for version 1

**DOI:** 10.3310/nihropenres.15267.r38150

**Published:** 2025-11-25

**Authors:** Boram Lee

**Affiliations:** 1 Ewha Womans University, Seodaemun-gu, South Korea

This manuscript is substantial, methodologically transparent, and addresses a highly important and timely topic in tobacco research. The authors attempt to review what has already done and what should be more investigated to fully understand the impacts of vaping policies. However, the manuscript is extremely long and difficulty to navigate, which significantly limits readability and accessibility.

 I recommend that the authors substantially shorten the manuscript by moving details to supplementary materials, or split it into 3-4 separate manuscript, each focusing on a distinct component. All figures are overwhelming and overly complex, making it difficult for readers to identify key messages. Additionally, providing a single summary table synthesizing the main findings would be helpful. At present, due to the length and complexity, it is extremely difficult to understand the core message and what the authors ultimately intent to convey.

Is the work clearly and accurately presented and does it cite the current literature?

Yes

If applicable, is the statistical analysis and its interpretation appropriate?

Not applicable

Are all the source data underlying the results available to ensure full reproducibility?

Yes

Is the study design appropriate and is the work technically sound?

No

Are the conclusions drawn adequately supported by the results?

Yes

Are sufficient details of methods and analysis provided to allow replication by others?

Yes

Reviewer Expertise:

tobacco policy, tobacco use, policy evaluation

I confirm that I have read this submission and believe that I have an appropriate level of expertise to state that I do not consider it to be of an acceptable scientific standard, for reasons outlined above.
